# Serum neurofilament light chain in multiple sclerosis: from biological signal to clinically informed decision-making

**DOI:** 10.3389/fneur.2026.1815022

**Published:** 2026-06-24

**Authors:** Ana Belén Caminero, Oscar Fernández

**Affiliations:** 1 Department of Neurology, Complejo Asistencial de Ávila, Ávila, Spain; 2 Institute of Biomedical Research of Malaga (IBIMA), Regional University Hospital of Malaga, Malaga, Spain; 3 Department of Pharmacology and Pediatrics, Faculty of Medicine, University of Malaga, Malaga, Spain

**Keywords:** biomarkers, disease monitoring, glial fibrillary acidic protein, implementation, MRI, multiple sclerosis, prognosis, serum neurofilament light chain

## Abstract

Serum neurofilament light chain (sNfL) is an analytically robust blood-based biomarker of recent neuroaxonal injury in multiple sclerosis (MS), supported by validated ultrasensitive immunoassays suitable for routine measurement. These assays now enable reliable measurement in routine care, and evidence links higher levels and rising trajectories to inflammatory activity, treatment response, and—at a population level—future tissue loss and disability risk. Clinical implementation is constrained by overinterpretation of single measurements: sNfL is not MS-specific, is strongly age-dependent, is influenced by systemic and neurological confounders, and reflects injury over weeks to months rather than cumulative neurodegeneration. This review proposes a clinically oriented framework in which sNfL serves as decision support alongside MRI and clinical assessment, not as a stand-alone trigger for therapy changes. We prioritize longitudinal interpretation anchored to an individual baseline or nadir, the use of age-adjusted reference frameworks (percentiles or Z-scores where available), standardization of key pre-analytical and analytical conditions, and structured approaches to discordance between biomarkers, imaging, and symptoms. We discuss the complementary role of serum glial fibrillary acidic protein (sGFAP) as a marker of chronic astroglial pathology and progression-dominant biology, and how combined biomarker patterns may refine interpretation when disability worsens despite limited inflammatory activity. Across the MS continuum—from prodromal states and radiologically isolated syndrome to relapsing and progressive phenotypes—we summarize practical use cases, limitations, and safety-critical “red flags” where marked sNfL surges warrant urgent reassessment. Finally, we highlight consensus-based implementation principles and research priorities, including pragmatic trials to test whether biomarker-informed strategies improve patient-relevant outcomes.

## 1 Introduction

Multiple sclerosis (MS) is a chronic inflammatory disease of the central nervous system (CNS) characterized by marked clinical heterogeneity and an unpredictable long-term course, in which inflammatory activity and neurodegenerative processes variably contribute to disability accumulation (1, 2). Despite major therapeutic advances and the widespread use of high-efficacy disease-modifying therapies (DMTs), long-term disability progression remains a central unmet need (3, 4).

Serum neurofilament light chain (sNfL) has emerged as one of the most extensively studied and analytically validated blood-based biomarkers of neuroaxonal injury in MS. The advent of ultrasensitive immunoassays has enabled reliable serum quantification, supporting longitudinal monitoring in both clinical trials and routine practice (5–7). Across observational cohorts and trial datasets, sNfL levels increase in association with inflammatory disease activity—including clinical relapses and MRI lesion formation—and typically decline following effective anti-inflammatory treatment. At a population level, higher or persistently elevated levels are also associated with subsequent tissue loss, disability accumulation, and worse long-term outcomes across MS phenotypes (8–12).

However, translating sNfL into clinical decision-making remains challenging. Interpretation requires careful consideration of biological variability, timing of measurement, analytical constraints, and contextual clinical factors, and should not rely on isolated values alone (13, 14). Rather than serving as a stand-alone biomarker, sNfL is best understood as a dynamic indicator of recent neuroaxonal injury that gains meaning when interpreted longitudinally and in conjunction with imaging and clinical assessment.

Guidance documents and systematic reviews have therefore proposed practical frameworks for the clinical implementation of sNfL in routine care (15, 16). These frameworks are particularly relevant because the clinical meaning of sNfL may vary across contexts, from inflammatory activity monitoring to more exploratory applications in progressive phenotypes and cognitive outcomes, where its added value over conventional MRI remains under investigation (17). Building on these considerations, additional international consensus initiatives—including those led by the Consortium of Multiple Sclerosis Centers (CMSC) and Delphi-based expert frameworks—have further emphasized longitudinal interpretation, age-adjusted contextualization, integration with MRI and clinical findings, and avoidance of rigid universal cut-offs in routine practice (18–20).

In parallel, complementary biomarkers such as serum glial fibrillary acidic protein (GFAP) are gaining increasing interest as potential indicators of astroglial pathology and progression-dominant biology, although their incremental clinical value remains under active investigation (11, 12).

**Figure 1:**
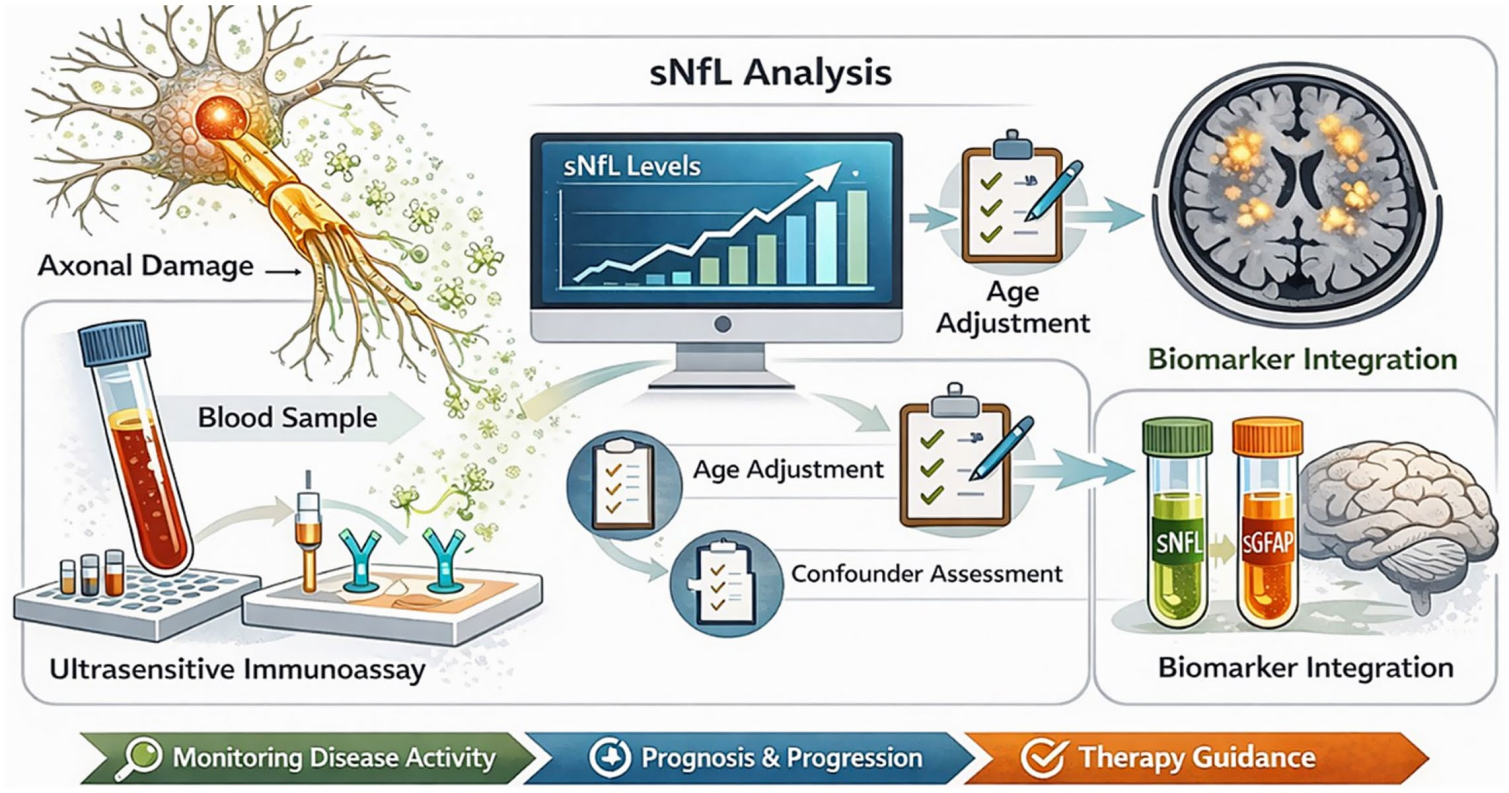
Conceptual framework for serum neurofilament light chain (sNfL) measurement and clinical interpretation in multiple sclerosis. Schematic overview of a pragmatic sNfL monitoring pathway in MS. After standardized sampling and ultrasensitive immunoassay measurement, results are interpreted using age adjustment and structured assessment of major confounders, prioritizing longitudinal change from an individual baseline/nadir and normative frameworks (percentiles/Z-scores when available) over absolute cut-offs. sNfL trajectories are then integrated with clinical assessment and MRI, optionally complemented by additional biomarkers (e.g., sGFAP), to support monitoring of disease activity, prognosis/progression, and therapy guidance while avoiding treatment changes based on isolated values.

Against this background, the present review provides a clinically oriented and biologically grounded framework for interpreting sNfL in contemporary MS care. Rather than reiterating biomarker validation studies, we focus on longitudinal trajectories, biological interpretation, and practical integration with imaging and complementary biomarkers, with particular attention to scenarios of clinical uncertainty, heterogeneous disease trajectories, and variability in real-world healthcare pathways where biomarker-informed interpretation may add value without becoming prescriptive (21). In practical terms, clinicians require an approach that distinguishes what is well established, what remains uncertain, and how biomarkers can realistically support bedside decision-making; this decision-support framework is operationalized in Figure 1, Box 1, and the implementation tables.

BOX 1 **Common pitfalls when using serum neurofilament light chain (sNfL) in MS—and how to mitigate them**

sNfL is a non-specific marker of recent neuroaxonal injury. Its clinical value increases when interpreted longitudinally, age-adjusted, and integrated with MRI and clinical context. The pitfalls below are the most frequent sources of misinterpretation and avoidable clinical overreaction.

- Pre-analytical and analytical pitfalls

Comparing absolute values across assays/platforms or laboratories.

*Mitigation:* Use the same assay/platform (ideally the same laboratory) for longitudinal follow-up. If a platform switch is unavoidable, document it and re-establish a new baseline.

Serum vs plasma and inconsistent sample handling (delay to centrifugation, repeated freeze–thaw, variable storage).

*Mitigation:* Standardize collection/processing; minimize freeze–thaw cycles; document specimen type and handling.

Single number mindset (treating sNfL as a stand-alone threshold).

*Mitigation:* Interpret trajectories (baseline/nadir → change over time) and use age-adjusted frameworks (percentiles/Z-scores when available).

- Timing and context pitfalls

Sampling too close to an inflammatory event (relapse/MRI activity) or too early after treatment initiation/switch.

*Mitigation:* Record timing relative to relapse/MRI and DMT change. Prefer repeat sampling to confirm a trend before reacting to a potentially transitional value.

Interpreting a late sample as “no activity” after a suspected event.

*Mitigation:* Pair sNfL with MRI and consider whether the sampling window may have missed the biological peak.

- Non-MS confounders (false positives)

Elevated sNfL due to non-MS causes (systemic infection/inflammation, trauma, stroke/TIA, seizures, other neurological disorders).

*Mitigation:* Screen for intercurrent illness/neurological events. If present, defer testing or interpret cautiously and prioritize clinical/MRI evaluation.

Renal dysfunction and other systemic factors associated with inflated values.

*Mitigation:* Document kidney function (normal vs. impaired). In chronic kidney disease, interpret with extra caution and rely more heavily on trajectories and concordant evidence.

Pregnancy/postpartum physiology or major lifestyle perturbations (e.g., extreme exertion).

*Mitigation:* Document pregnancy/postpartum status and lifestyle extremes; avoid using isolated results to drive high-stakes decisions.

- MS-specific interpretation pitfalls

Low/normal sNfL = no disease activity.

*Mitigation:* Do not down-triage inflammatory disease activity based on low sNfL. MRI and clinical findings remain primary for localization and acute management.

High sNfL = relapse (etiological overinterpretation).

*Mitigation:* Treat high sNfL as an alarm signal prompting structured reassessment, not as a diagnosis.

Missing progression biology (PIRA/progression) when sNfL is low.

*Mitigation:* In disability progression with low sNfL, consider progression-dominant mechanisms and add complementary assessment (e.g., sGFAP, quantitative MRI/spinal cord metrics, OCT where available).

- Action pitfalls

Escalating/switching therapy based on a single elevated sNfL in an otherwise stable patient.

*Mitigation:* Confirm with repeat testing (standardized timing, same platform), reassess confounders, and integrate MRI/clinical status before major treatment changes.

Ignoring a confirmed upward trend because MRI is “stable.”

*Mitigation:* Rising/persistently high sNfL with stable MRI should trigger tiered actions: verify validity → repeat → consider earlier MRI and broader evaluation (including spinal cord and alternative etiologies).

## 2 Methods and scope

This narrative review was developed to provide an implementation-focused framework for interpreting serum neurofilament light chain (sNfL) in multiple sclerosis (MS) in conjunction with clinical assessment and MRI, with targeted consideration of serum glial fibrillary acidic protein (sGFAP) where relevant. A structured literature search (last updated February 2026) was conducted in PubMed/MEDLINE and complemented by searches in Embase and Scopus, alongside manual screening of reference lists from key reviews, meta-analyses, and consensus/guidance documents. Search terms combined “multiple sclerosis” and “neurofilament light”/“NfL,” with modifiers including “serum”/“plasma,” assay platforms (e.g., Simoa, Lumipulse, CLIA/ECLIA), and implementation-relevant concepts (percentile/Z-score, confounders, pregnancy, renal function, clinical utility). Study selection was guided by relevance to clinical interpretation and real-world implementation. Evidence was prioritized when derived from independent cohorts, longitudinal studies, randomized trials and *post hoc* trial biomarker analyses, meta-analyses, and clinically applicable real-world datasets. When multiple publications reported overlapping cohorts, we preferentially cited the most comprehensive or most recent analysis and avoided double-counting conclusions. The synthesis is structured around biological/analytical foundations, principles of clinical interpretation (including confounder management and discordance), and pragmatic use cases across the MS continuum, culminating in consensus-aligned implementation recommendations and research priorities.

## 3 Biological meaning of sNfL and GFAP: what do they really measure?

### 3.1 Neuroaxonal injury versus neurodegeneration

Neurofilaments are class IV intermediate filaments that form a core structural scaffold of the neuronal cytoskeleton, particularly enriched in large, myelinated axons. They assemble as heteropolymers of light (NfL), medium (NfM), and heavy (NfH) subunits and contribute to axonal caliber, mechanical stability, and conduction properties (22, 23).

NfL is the most abundant and relatively soluble subunit. Its preferential expression in long-projecting axons and its molecular stability make it especially prone to release after axonal structural disruption, regardless of the underlying pathological mechanism (13, 24, 25). This biology explains why serum NfL (sNfL) acts as a quantitative marker of neuroaxonal injury rather than a disease-specific biomarker. A key interpretative point is that sNfL should not be conflated with cumulative neurodegeneration. Biologically, sNfL reflects the rate and magnitude of recent axonal injury over weeks to months, not the irreversible neuronal loss accumulated over years (8, 26, 27). This distinction is supported by consistent associations at the population level, while its value for individual-level prediction remains more limited.

In MS, inflammatory demyelination with associated axonal transection is a dominant driver of sNfL elevation, both during overt relapses and during subclinical MRI activity (28). This temporal and biological specificity has direct clinical implications. Patients with advanced MS can exhibit substantial brain atrophy or cortical thinning while maintaining age-adjusted sNfL within reference ranges if inflammatory activity is minimal. Conversely, in early disease stages, pronounced sNfL elevations may occur despite preserved brain volume, reflecting active axonal injury that can precede measurable tissue loss (8, 29, 30).

### 3.2 GFAP as a marker of chronic astroglial pathology

Glial fibrillary acidic protein (GFAP) is the principal intermediate filament of mature astrocytes and a key structural component of the astrocytic cytoskeleton. Astrocytes support CNS homeostasis through metabolic coupling, synaptic regulation, blood–brain barrier maintenance, and immune signalling (31–34). Astrocytic activation and injury increase GFAP expression and release into CSF and blood. In contrast to sNfL, circulating GFAP is thought to more consistently reflect chronic astroglial activation and aspects of tissue remodelling than acute focal axonal injury, although its specificity and temporal dynamics remain less well defined than those of sNfL (27, 35). In MS, serum GFAP (sGFAP) has shown more consistent associations at the cohort level with progressive disease stages, grey matter pathology, and disability accumulation than with acute inflammatory activity (36–38). However, most of these associations are derived from cohort-level analyses, and their translation into individual-level clinical decision-making remains an area of ongoing investigation.

### 3.3 Biological complementarity of sNfL and sGFAP

From a pathophysiological perspective, sNfL and sGFAP capture distinct but complementary biological domains. sNfL primarily indexes acute or subacute neuroaxonal injury that is often inflammation-driven, whereas sGFAP more closely reflects chronic astroglial activation and tissue remodelling, processes increasingly implicated in progression independent of relapse activity (PIRA) (27, 30, 37).

Accordingly, a weak cross-sectional relationship between sNfL and structural measures of neurodegeneration should not be viewed as a limitation of sNfL; rather, it highlights that these biomarkers reflect different temporal scales and biological substrates (8, 10). Interpreting sNfL and sGFAP together—and in relation to imaging measures that capture focal inflammation (lesions) and accumulated tissue loss (atrophy)—can provide a more biologically faithful representation of MS activity and progression risk than reliance on any single biomarker (Table 1) (39–46).

**Table 1:** Biological domains captured by circulating and imaging biomarkers in multiple sclerosis (39–46).

Biomarker/Tool	Primary biological process	Temporal scale	Clinical meaning	Key references
sNfL	Recent neuroaxonal injury	Weeks–months	Quantifies rate and magnitude of recent axonal damage, mainly inflammation-driven	(7, 9, 10, 13)
sGFAP	Astroglial activation, gliosis	Months–years	Reflects chronic tissue remodelling and progression-related biology	(11, 12, 36–38)
MRI (lesions)	Focal inflammation	Days–weeks	Detects and localizes inflammatory activity	(39–41)
MRI (atrophy)	Accumulated tissue loss	Years	Structural footprint of cumulative damage	(8, 42–44)
OCT	Retinal neuroaxonal loss	Years	Marker of cumulative neurodegeneration	(4, 45)
Integrated biomarker approaches	Multimodal disease characterization	Variable	Combines fluid and imaging biomarkers to improve biological interpretation	(46)

## 4 Analytical and physiological constraints: what limits interpretation

### 4.1 Analytical platforms and non-interchangeability of values

Meaningful clinical interpretation of serum neurofilament light chain (sNfL) depends not only on analytical sensitivity and precision, but also—crucially—on longitudinal stability within a given measurement system. Early ELISA-based methods enabled reliable quantification in cerebrospinal fluid but generally lacked the sensitivity required for serum, where concentrations are markedly lower (5). The subsequent development of ultrasensitive immunoassays, including single-molecule digital approaches such as Simoa, represented a major step forward by enabling reliable quantification of NfL in blood (47, 48). More recently, automated chemiluminescent (CLIA), electrochemiluminescent (ECLIA), and digital immunoassay platforms have facilitated broader clinical implementation and improved laboratory workflow integration, although analytical challenges may persist in the lower concentration range typically observed in clinically stable MS (49–51).

These analytical advances should not be interpreted as implying that one platform is universally superior for all clinical purposes. In routine MS care, assay selection also depends on automation, reproducibility, inter-laboratory performance, availability, cost, and analytical performance within the concentration range most relevant to longitudinal follow-up (51–53). In this context, chemiluminescent and electrochemiluminescent platforms may be particularly well suited to routine laboratory workflows, even if analytical sensitivity at very low concentrations tends to be greater with digital assay platforms (51–55).

Despite these advances, multiple platforms currently coexist, differing in antibody pairs, calibration strategies, and reference materials. Although cross-platform correlations are often high, systematic proportional bias and constant offsets are consistently observed, particularly in the lower concentration range most relevant for longitudinal MS monitoring (14, 51–55). As a result, absolute sNfL values are not interchangeable across platforms. A mid-follow-up switch in assay or laboratory can therefore falsely mimic biological change and undermine interpretation unless the transition is explicitly managed, for example by documenting the switch and re-establishing a new baseline (14, 51, 55). Taken together, the key analytical principle for longitudinal monitoring is to ensure within-platform consistency interpret results in relation to the characteristics of the assay used, and avoid direct comparison of absolute values across methods (Table 2).

**Table 2:** Serum NfL analytical platforms: clinically relevant comparison for neurologists.

Platform category	Representative assays	What this platform offers	Main limitation for clinical follow-up	Are absolute values interchangeable across platforms for clinical follow-up?
High-sensitivity ELISA	Research ELISA kits	Historical first method; low cost	Insufficient sensitivity in serum; floor effects at low concentrations	No (5, 52)
ECLIA	Elecsys NfL	Robust automation; well suited for routine laboratory integration	Systematic scale differences vs. other platforms	No (49, 51, 53)
Automated CLIA	Lumipulse G NfL blood	Good precision; high-throughput clinical workflow	Concentration-dependent bias vs. digital assays	No (51–53)
Microfluidic CLIA	Ella™	Rapid turnaround; preserved longitudinal trajectories	Systematic bias vs. Simoa	No (53, 55)
Digital immunoassay	Simoa NF-light/automated digital assays	Highest analytical sensitivity; strong performance in the low concentration range	Cost and availability; harmonization still evolving, clinical added value in stable disease remains context-dependent	Not directly interchangeable; systematic offsets observed (14, 47–55)

### 4.2 Physiological modifiers and normative reference frameworks

Beyond analytical variability, sNfL concentrations are shaped by physiological and systemic modifiers that can confound interpretation if not explicitly considered. Age is the dominant determinant, with non-linear increases across the lifespan and an accelerated rise beyond approximately 50 years—making fixed absolute cut-offs inherently misleading (56–60). Importantly, age-related increases are accompanied by greater interindividual variability in older populations, reinforcing the need for age-adjusted percentiles or Z-score–based interpretation rather than reliance on universal thresholds (56–60).

Higher body mass index is associated with lower measured sNfL concentrations, an effect thought to reflect hemodilution and distribution-volume differences rather than reduced neuroaxonal injury, and BMI is increasingly incorporated into refined normative models and individualized interpretative frameworks (57, 59, 60). Renal dysfunction is consistently associated with higher circulating sNfL levels, most plausibly reflecting reduced systemic clearance rather than increased neuroaxonal injury, particularly in older adults and patients with chronic kidney disease (58–62).

Other modifiers relevant in practice include pregnancy and the postpartum period, intense physical exertion, and circadian variation, as well as multisystem conditions associated with accelerated biological aging (13, 56). Because these factors can shift sNfL independently of MS activity, failure to document timing and context can lead to misinterpretation—particularly when clinicians react to isolated values rather than confirmed trajectories.

Importantly, sNfL is not disease-specific and should be interpreted in light of relevant confounders and differential diagnoses. In addition to physiological modifiers such as age, BMI, and renal function, vascular and systemic comorbidity may contribute to higher circulating sNfL values independent of MS activity. Elevated concentrations have also been described in acute infection, cerebrovascular disease, traumatic brain injury, seizures, and coexisting neurodegenerative disorders including Alzheimer’s disease and amyotrophic lateral sclerosis, underscoring that isolated elevations cannot be attributed to MS activity per se (59–62).

Timing is equally important. sNfL should be viewed as a delayed integrative marker of recent neuroaxonal injury over weeks to months rather than a real-time activity marker. Acute events such as relapse, stroke, trauma, or systemic inflammation may produce transient increases, whereas chronic neurodegenerative processes may lead to more sustained elevations. Interpretation should therefore remain longitudinal and context-dependent, integrating age-adjusted reference frameworks, BMI and renal function when relevant, comorbidities, and the clinical/MRI time course (59–61) (Table 3).

**Table 3:** Major physiological, systemic, and neurological confounders relevant to serum sNfL interpretation in clinical practice.

Category	Confounder	Direction of effect	Typical magnitude	Key clinical implication	Key references
Physiological	Age	↑	Progressive, non-linear; steeper >50 years	Mandatory age-adjusted interpretation (percentiles or Z-scores); absolute cut-offs misleading	(9, 56)
Physiological	Body mass index (BMI)	↓	Moderate*	Hemodilution effect; consider BMI-adjusted models where available	(57)
Physiological	Diurnal variation	↕	~5–10%	Standardize sampling time for longitudinal monitoring	(56)
Physiological	Intense physical exercise	↑ (transient)	Up to 24–48 h	Avoid sampling shortly after strenuous exercise	(13)
Physiological	Pregnancy	↓	Mild*	Hemodilution and immunological modulation; interpret longitudinally	(13, 56)
Physiological	Postpartum period	↑ (transient)	Mild–moderate*	Elevations usually associated with relapses or inflammatory reactivation	(13, 56)
Systemic	Renal dysfunction	↑	~20–80%	Reduced systemic clearance; adjust interpretation in CKD	(58, 62)
Neurological (acute)	Ischemic or hemorrhagic stroke	↑↑↑	20–50×	Reflects massive axonal injury; unrelated to MS activity	(13, 24)
Neurological (acute)	Traumatic brain injury	↑↑↑	Severity-dependent	Major confounder; temporal correlation essential	(9, 13)
Neurological (acute)	Seizures/status epilepticus	↑↑	Up to ~5×	Elevation may mimic inflammatory activity; timing critical	(13)
Neurodegenerative	Amyotrophic lateral sclerosis (ALS)	↑↑↑	Often >150–300 pg./mL	Diagnostic and prognostic relevance; orders of magnitude higher than MS	(59)
Neurodegenerative	Alzheimer’s disease	↑	Mild–moderate*	Chronic neurodegeneration; persistent elevation	(35, 59)
Neurodegenerative	Frontotemporal dementia/atypical parkinsonism	↑	Moderate*	Reflects ongoing neuroaxonal loss	(24, 59)
Systemic	Sepsis/acute systemic inflammation	↑	Mild–moderate*	Exclude systemic illness before attributing to MS	(59, 61)

*Magnitude descriptors are indicative and context-dependent. In general terms: *mild* ≈ slight increase above expected age-adjusted range (often within ~1–2×), *moderate* ≈ clear elevation above expected range (commonly ~2–3×), *marked/severe* ≈ substantially elevated levels (often >3–10×, particularly in acute neurological injury). These ranges are approximate and may vary depending on the underlying condition and clinical context. They should not be interpreted as fixed thresholds. Clinical interpretation should prioritize longitudinal trajectories and integration with clinical and radiological findings.

Taken together, these constraints explain why age- (and increasingly BMI-) adjusted percentiles or Z-scores provide a more clinically meaningful interpretative framework than raw absolute concentrations. In particular, values positioned at the upper end of the age-adjusted reference distribution may carry greater prognostic relevance than absolute concentrations alone, reinforcing the value of percentile- and Z-score–based contextualization for individual risk assessment (10, 15, 57). Normative approaches generally outperform absolute values for predicting inflammatory activity and treatment response, but they remain assay-specific and cannot be transferred across platforms. This reinforces a central implementation principle: results should be interpreted longitudinally within the same analytical framework and contextualized using appropriate normative references (10, 14, 15).

**Figure 2:**
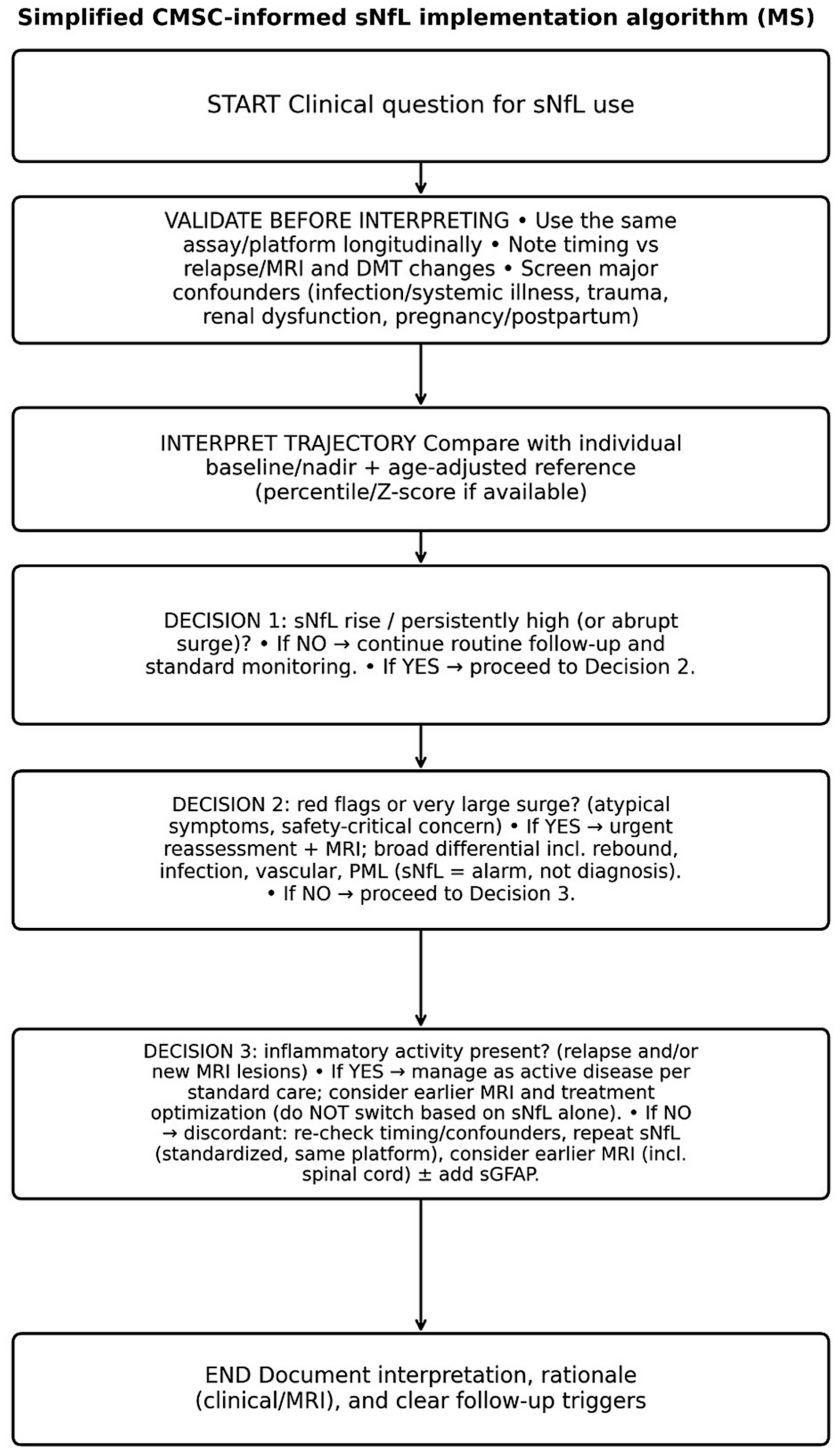
One-column, implementation-focused algorithm for using serum neurofilament light chain (sNfL) in multiple sclerosis (CMSC-informed). This one-column flowchart outlines a clinic-ready approach to using sNfL as decision support alongside clinical assessment and MRI. It emphasizes: (1) Validate before interpreting—use the same assay/platform longitudinally; account for sampling timing relative to recent relapse/MRI activity and DMT changes; and screen major confounders (systemic infection/inflammation, trauma, renal dysfunction, pregnancy/postpartum); (2) Interpret trajectories—compare with the patient’s baseline/nadir and age-adjusted reference frameworks (percentiles/*Z*-scores when available); and (3) Act in tiers via three sequential decisions—whether sNfL is confirmed rising/persistently high (or abruptly surging), whether red flags indicate a safety-critical scenario requiring urgent reassessment and MRI, and whether inflammatory activity is corroborated (relapse and/or new MRI lesions). In discordant cases, reassess timing/confounders, confirm the trend with repeat standardized testing on the same platform, and consider earlier MRI (including spinal cord when relevant) and complementary biomarkers (e.g., sGFAP) to tailor monitoring intensity. The algorithm discourages switching therapy based on sNfL alone and ends with documentation of interpretation and clear follow-up triggers; future frameworks may incorporate validated MRI + sNfL data-driven outputs to further personalize actions.

Figure 2 illustrates the conceptual framework for serum neurofilament light chain (sNfL) measurement and clinical interpretation in multiple sclerosis.

## 5 sNfL as a marker of inflammatory activity in MS

Inflammatory activity in multiple sclerosis (MS) is driven by focal immune-mediated demyelination with variable degrees of acute neuroaxonal injury. Clinical relapses and MRI lesions remain the core measures of inflammatory disease activity, yet both incompletely capture the biological burden of tissue injury—particularly when activity is subclinical, anatomically restricted, or temporally dispersed. Serum neurofilament light chain (sNfL) provides a sensitive and quantitative readout of inflammation-related neuroaxonal damage and consistently associates with clinical and radiological activity across cohorts and trials (6, 8).

Across observational studies and randomized datasets, sNfL rises during periods of inflammatory activity and declines following effective disease-modifying therapy (DMT), supporting its role as a dynamic marker of recent axonal injury (7, 10). Importantly, sNfL is not a binary “activity test,” but reflects the downstream consequence of inflammation—axonal injury—over a biologically delayed window of weeks to months. MRI therefore remains essential to detect and localize focal inflammatory activity, while sNfL complements MRI by quantifying the biological severity of injury and capturing activity that may be clinically silent or not fully appreciated on routine imaging (15). Key interpretive relationships between sNfL, relapses, and MRI inflammatory activity are summarized in Table 4.

**Table 4:** Clinical interpretation of sNfL in relation to inflammatory activity in multiple sclerosis.

Clinical scenario	Expected sNfL behavior	Clinical value	Key limitations	Key references
Acute clinical relapse	Delayed rise, peak at ~4–8 weeks	Confirms recent inflammatory axonal injury	Not a real-time marker; timing dependent	(6–8)
New Gd + MRI lesions	Moderate increase, burden-dependent	Integrates cumulative lesion load	Limited sensitivity for isolated focal lesions	(6–8, 15)
High inflammatory burden	Sustained or recurrent elevation	Supports identification of biologically active, high-risk disease	Requires longitudinal assessment	(6–10)
Low sNfL values	Stable low levels over time	High negative predictive value for substantial inflammatory activity	Does not exclude isolated lesions	(10, 15)
After DMT initiation	Progressive decline over weeks–months	Marker of biological treatment response	Assay-, timing-, and baseline-dependent	(6, 7, 10, 57)

### 5.1 Temporal dynamics in relation to clinical relapses

Longitudinal studies show a reproducible temporal pattern: sNfL typically rises around relapse-associated inflammatory injury and then declines over subsequent weeks to months, consistent with a biologically delayed and time-integrated signal of recent neuroaxonal damage (6, 7). Beyond documenting recent injury, higher baseline sNfL values and—more importantly—sustained upward trajectories are associated with an increased probability of near-term relapse, supporting sNfL as a short-horizon risk marker when interpreted longitudinally and within age-adjusted reference frameworks (63).

### 5.2 Relationship with MRI inflammatory activity

Because a substantial proportion of MS inflammatory activity is clinically silent, the relationship between sNfL and MRI activity is central to interpretation. Across longitudinal datasets, sNfL concentrations are higher in patients with gadolinium-enhancing lesions and/or new or enlarging T2 lesions than in radiologically inactive patients (6, 15). A dose–response relationship is consistently reported, with higher sNfL levels accompanying greater lesion number and volume (8, 63).

High-frequency imaging studies suggest that sNfL peaks often follow the appearance of new enhancing lesions by approximately 1–3 months, reinforcing that sNfL reflects recent inflammatory injury rather than contemporaneous MRI activity (64, 65). Additional studies further support associations between sNfL, blood–brain barrier disruption, inflammatory lesion activity, and early inflammatory disease processes following demyelinating events (66, 67).

Low sNfL values can be informative at a probabilistic level: low age-adjusted percentiles are associated with a low likelihood of substantial recent inflammatory burden. However, sensitivity is limited for isolated, small, or anatomically restricted lesions, meaning that low sNfL cannot replace MRI for detecting focal inflammatory activity (66, 67).

### 5.3 Magnitude of sNfL response and sensitivity limitations

The magnitude of sNfL elevation broadly reflects the biological intensity of inflammatory neuroaxonal injury. More extensive inflammatory events—multifocal relapses, large or prolonged enhancing lesions, and spinal cord involvement—tend to produce higher sNfL peaks than mild or localized events (8, 30). Anatomical location also modulates the response: extensive myelitis or brainstem involvement often yields larger increases than isolated optic neuritis, although optic neuritis can still be associated with substantial elevations when there is concurrent inflammatory burden elsewhere (30).

At the individual-event level, sNfL has incomplete sensitivity. Mild relapses or limited MRI activity may not produce marked elevations, particularly when sampling falls outside the optimal kinetic window. Interindividual variability in axonal vulnerability and baseline physiology further contributes to heterogeneous responses (66, 68). Clinically, this implies an asymmetric interpretation: normal/low sNfL does not exclude inflammatory activity, whereas marked elevations strongly support recent neuroaxonal injury, albeit not etiologically specific to MS.

### 5.4 Clinical performance and integration into practice

The practical value of sNfL depends on inflammatory burden, sampling timing, and the interpretative framework used. High age-adjusted percentile or Z-score ranges provide high specificity for recent inflammatory activity but only modest sensitivity, particularly in low-burden disease. Performance improves substantially when sNfL is interpreted longitudinally, with confirmation of sustained changes over time (10, 15).

From an implementation standpoint, sNfL should be framed as a complementary biomarker reflecting recent cumulative inflammatory axonal injury over roughly the prior 2–3 months. It can support decisions on monitoring intensity, prompt targeted reassessment when discordant with clinical or MRI findings, and help contextualize disease activity over time. However, it does not replace MRI or clinical evaluation.

## 6 Prognostic value of sNfL: disability accumulation and progression biology

Beyond its established role as a marker of acute inflammatory activity, serum neurofilament light chain (sNfL) provides clinically relevant prognostic information in multiple sclerosis (MS) when interpreted longitudinally and within an appropriate biological framework. Importantly, sNfL does not predict future disability in a deterministic or linear manner. Rather, it reflects the recent burden and intensity of neuroaxonal injury, which probabilistically increases the likelihood of subsequent tissue loss and functional decline over longer time horizons.

A consistent body of longitudinal cohort studies and *post hoc* analyses of clinical trials demonstrates that higher sNfL levels—particularly when sustained or recurrent over time—are associated with an increased risk of disability accumulation, accelerated brain and spinal cord atrophy, and worse long-term outcomes across MS phenotypes (8–10, 15, 69). Prognostic performance is strongest when sNfL is interpreted longitudinally as a biological trajectory (direction, persistence, and magnitude of change) and when integrated with baseline disability measures, imaging, and complementary biomarkers such as sGFAP (10, 69, 70).

### 6.1 Relapse-associated disability worsening and inflammatory neuroaxonal injury

The strongest and most reproducible prognostic associations of sNfL relate to relapse-associated disability worsening (RAW). Elevated baseline sNfL values and, more importantly, persistently high trajectories identify patients at increased risk of confirmed disability worsening (CDW), earlier attainment of Expanded Disability Status Scale (EDSS) milestones, and greater long-term tissue loss and disability accumulation, even after adjustment for relapse frequency and MRI lesion burden (8, 10, 71, 72).

Patients with recurrent sNfL peaks or sustained elevations show consistently worse outcomes than those with transient increases followed by durable suppression, whereas persistently low sNfL trajectories are associated with a lower probability of near-term disability accumulation—even among patients with similar clinical presentations at baseline (9, 72). This pattern is clinically intuitive: repeated or incompletely suppressed inflammatory neuroaxonal injury increases the likelihood that irreversible tissue loss will accrue over time, translating into higher risk of relapse-linked stepwise disability.

Multivariable prognostic models incorporating sNfL alongside age, baseline disability, and MRI metrics consistently outperform traditional clinical–radiological predictors, supporting the use of sNfL as an adjunctive prognostic biomarker that refines risk stratification (10).

### 6.2 Limits of sNfL for progression independent of relapse activity (PIRA)

Despite its prognostic value for relapse-associated outcomes, sNfL has important limitations for capturing progression independent of relapse activity (PIRA). Once overt inflammatory activity is excluded, associations between sNfL and disability progression attenuate substantially. Comparative studies demonstrate that astroglial biomarkers—particularly serum glial fibrillary acidic protein (sGFAP)—show stronger and more consistent associations with progression, cortical/grey matter atrophy, and long-term disability than sNfL (36, 37, 73).

This biological dissociation reflects distinct pathological domains. sNfL primarily captures recent or subacute axonal injury (often inflammation-driven), whereas GFAP reflects chronic astroglial activation and tissue remodelling—processes more closely linked to slow progression and PIRA. Consequently, normal or low sNfL values do not exclude ongoing progression, particularly in later disease stages or progressive phenotypes (70, 74). In practice, this is a key interpretive safeguard: a “quiet” sNfL profile can coexist with clinically meaningful progression, especially when structural damage is advanced or progression biology predominates (Table 5).

**Table 5:** The complementary prognostic roles of sNfL and sGFAP across disability domains (RAW vs. PIRA; atrophy patterns; typical clinical context).

Disability domain/outcome	sNfL: biological signal	Strength of evidence	sGFAP: biological signal	Clinical interpretation	Key references
Relapse-associated disability worsening (RAW)	Reflects magnitude and frequency of recent inflammatory axonal injury; elevated baseline or recurrent peaks predict higher risk of CDW	Strong, consistent across cohorts and trials	Secondary or modest association	High or rising sNfL identifies patients at increased risk of relapse-related disability and supports closer monitoring or treatment intensification	(8–10, 69, 71, 72)
Short- to mid-term disability accumulation	Sustained elevations integrate cumulative neuroaxonal injury burden over months–years	Moderate–strong, enhanced by longitudinal analysis	Complementary, weaker than sNfL in early stages	Longitudinal trajectories outperform single values; persistent elevation signals higher biological aggressiveness	(8–10, 69)
Progression independent of relapse activity (PIRA)	Limited sensitivity once overt inflammation is excluded; associations attenuate after adjustment	Weak–moderate	Stronger association with progression-related pathology and disability worsening	Normal sNfL does not exclude progression; GFAP may provide a more informative signal for PIRA-related biology	(36, 37, 70, 73, 74)
Grey matter damage and cortical atrophy	Weak or inconsistent cross-sectional associations	Weak	Stronger and more consistent association	sNfL insufficient to monitor grey matter–driven disability; GFAP adds critical information	(36, 37, 73)
Silent progression and cognitive decline	Poor sensitivity; often normal despite ongoing decline	Weak	Moderate association with cognitive impairment and grey matter pathology	Absence of sNfL elevation should not reassure; multimodal assessment required	(17, 36, 37)
Early disease biological risk stratification	Elevated or rising trajectories predict aggressive inflammatory courses	Strong	Adds prognostic refinement	Combined biomarker profiling improves early risk stratification beyond clinical–MRI markers	(10, 57, 67, 69)
Late-stage disease with advanced atrophy	Often low or age-expected despite disability	Limited utility	Frequently elevated, reflecting chronic gliosis	Low sNfL reflects reduced inflammatory injury, not disease quiescence	(36, 37, 70, 73, 74)

### 6.3 Individual biological trajectories and clinical implications

Combined biomarker approaches provide improved prognostic resolution by capturing complementary biological domains in MS. Joint assessment of sNfL and sGFAP improves prediction of long-term disability accumulation and helps discriminate between predominantly inflammatory trajectories and progression-dominated disease biology (37, 75–77). Conceptually, sNfL reflects recent injury load, while sGFAP adds information about chronic glial pathology and remodeling—processes that may diverge substantially within the same patient and across disease stages (78).

Structural measures further contextualize biomarker interpretation. Quantitative MRI and optical coherence tomography reflect accumulated anatomical damage over years, whereas sNfL reflects neuroaxonal injury occurring over weeks to months. This temporal dissociation explains the frequent lack of strong cross-sectional correlations between fluid biomarkers and structural metrics (8, 10). Clinically, rising sNfL trajectories in the presence of limited atrophy suggest a potentially modifiable phase of active tissue injury, whereas advanced atrophy with low sNfL—often accompanied by elevated sGFAP—suggests later-stage biology that is less responsive to purely anti-inflammatory intervention.

Early combined biomarker profiling also highlights substantial interindividual heterogeneity in MS biology. In a large multicentre cohort, baseline sNfL and sGFAP measured within the first year of disease independently predicted relapse-associated worsening, PIRA, and attainment of EDSS 3 over more than 6 years of follow-up (77). These findings reinforce that patients with similar clinical phenotypes may follow divergent biological trajectories and long-term outcomes.

From a clinical perspective, these data support a trajectory-based, multimodal interpretation. Persistently elevated or rising sNfL identifies patients with higher-risk inflammatory biology and greater probability of relapse-associated disability, whereas low and stable sNfL trajectories—particularly when accompanied by elevated sGFAP—are more consistent with progression-dominated disease. Multimodal frameworks integrating sNfL, sGFAP, structural imaging, and clinical variables can improve prognostic stratification and support contextual decision-making, without implying deterministic prediction or prescriptive treatment rules (10, 15, 19) (Table 6).

**Table 6:** Practical prognostic interpretation matrix for combined sNfL/sGFAP trajectories (low–stable vs rising/persistent; suggested clinical meaning and recommended integration with MRI and disability measures).

Pattern (trajectory-based)	Most likely biological interpretation	Suggested actions (proportional, CMSC-aligned)	Key pitfalls/checks	Key references
High/rising sNfL + low/age-expected sGFAP	Inflammation-dominant neuroaxonal injury (recent inflammatory activity or incomplete suppression on therapy) (19).	Confirm timing and confounders; integrate MRI (new/enlarging T2, Gd+); consider earlier MRI and treatment optimization/escalation supported by overall evidence.	Avoid attributing to MS if intercurrent illness/trauma; verify same assay/platform; consider repeat to confirm trend.	(6–10, 15, 19)
High/rising sNfL + high/rising sGFAP	Mixed inflammatory and progression-related biology: (19, 70).	Treat as higher-risk state: expedited clinical review and MRI; confirm trajectory with repeat testing; consider therapy optimization and close monitoring (quantitative MRI/atrophy where available).	Non-specific elevations (e.g., vascular, infection) can raise both; ensure differential diagnosis and safety work-up if atypical features.	(12, 36, 37, 70, 73–77)
Low/age-expected sNfL + high/rising sGFAP (especially with disability worsening)	Progression-dominant biology/PIRA-like worsening more likely than focal inflammatory activity (26, 110).	Emphasize progression-oriented assessment: quantitative MRI (atrophy/spinal cord), neuroperformance/OCT if available; optimize symptomatic and comorbidity management; consider progression-focused strategy rather than reflex inflammatory escalation.	Do not ‘diagnose progression’ from sGFAP alone; assay harmonization and normative frameworks are still evolving—use longitudinal interpretation.	(36, 37, 70, 73, 74, 77)
Low/age-expected sNfL + low/age-expected sGFAP	Low biomarker evidence of recent injury; stable biology more likely (if concordant with clinical/MRI) (19).	Continue routine follow-up; use standard clinical/MRI monitoring; repeat biomarkers only if clinical question arises or as part of structured monitoring.	Beware false reassurance: MRI/clinical findings remain primary; timing may miss a transient peak.	(15, 17, 19)

## 7 sNfL as a pharmacodynamic and monitoring biomarker under disease-modifying therapies

The availability of ultrasensitive immunoassays has enabled a more precise characterization of disease-modifying therapy (DMT) effects on serum neurofilament light chain (sNfL). In this setting, sNfL functions primarily as a pharmacodynamic readout of inflammation-associated neuroaxonal injury: effective anti-inflammatory therapy is generally followed by declining sNfL levels, whereas persistent elevation or renewed increase may indicate residual or recurrent injury (7, 10, 15).

Across randomized trials and real-world cohorts, three clinically relevant principles emerge (7, 10, 15):

- On-treatment decreases track suppression of inflammatory activity. Effective DMT initiation is typically followed by a reduction in sNfL over the subsequent months, with larger and earlier declines generally seen in patients with higher pre-treatment inflammatory activity and under more potent anti-inflammatory therapies.
- Interpretation should be based on within-patient change rather than isolated values, taking into account age-adjusted reference frameworks, relapse or MRI timing, recent steroid use, and assay/platform consistency.
- Persistent elevation or confirmed re-elevation on treatment should prompt structured reassessment—clinical review, MRI, adherence/exposure evaluation, and exclusion of non-MS confounders—rather than automatic escalation.

### 7.1 Time to effect and expected trajectories under treatment

Under effective therapy, biologically meaningful reductions in sNfL are typically detectable within weeks to months and tend to consolidate over 6–12 months in the absence of further inflammatory activity (7, 64, 65). Early post-treatment measurements may still reflect pre-treatment events or subclinical MRI activity preceding therapeutic suppression. Conversely, delayed normalization, plateauing above age-expected ranges, or secondary rises after initial decline suggest ongoing or recurrent neuroaxonal injury and warrant contextual reassessment rather than reflexive action. In practice, the interpretive focus is less on achieving an “absolute normal value” and more on whether the trajectory shows durable suppression consistent with sustained inflammatory control (Table 7).

**Table 7:** Suggested monitoring timepoints and expected sNfL kinetics after DMT initiation (interpretation by phase: early, consolidation, maintenance).

Monitoring phase	Suggested timepoint (relative to DMT start)	Purpose (what this sample “answers”)	Expected sNfL pattern if inflammatory injury is being suppressed	If sNfL is elevated or rising (confirmed)	Practical notes/caveats	Key references
Pre-treatment baseline	0 (or within 0–4 weeks before start)	Establish individual baseline and pre-treatment injury burden	May be high if recent relapse/MRI activity; not a “steady-state” marker	High baseline may increase the probability of near-term peaks even after starting DMT	Document relapse/MRI activity in prior 2–3 months; sample before steroids if possible; record confounders (infection, trauma, renal dysfunction, postpartum, etc.)	(6–8, 10, 24, 56, 80)
Early “carryover” window	4–8 weeks *(optional; useful in highly active disease or after switch)*	Detect early trajectory direction and identify obvious biological non-control	Often still reflects pre-treatment activity; decline may begin in rapid-acting/high-efficacy therapies	Persistent marked elevation can indicate ongoing activity, but also timing carryover	Avoid overreacting to a single early value; interpret strictly vs. baseline and context; same platform/lab essential	(6, 7, 10, 80)
Early response checkpoint	~3 months (10–14 weeks)	Assess whether suppression is emerging as expected	Meaningful decline expected in many responders; larger/earlier declines with highly effective anti-inflammatory DMTs	If unchanged/high: reassess timing (recent relapse/MRI lesions), adherence/exposure, confounders; consider earlier MRI if concern persists	Best interpreted as change-from-baseline (or age-adjusted Z/percentile change) rather than absolute value	(6, 7, 10, 57, 80)
Consolidation checkpoint	~6 months	Confirm consolidation of biological suppression	Further decline/near-nadir if no new inflammatory activity; trajectories should stabilize	Plateau above age-expected ranges or upward drift suggests residual activity, delayed normalization, or alternative injury	Good time to align with MRI review cadence; confirm reproducibility with repeat if unexpected	(6, 7, 10, 57, 80)
Stability confirmation (“new baseline/nadir”)	~12 months	Define on-treatment nadir/durable suppression	Stable low/age-expected trajectory in well-controlled inflammatory disease	Persistent elevation or re-elevation strengthens case for structured reassessment (MRI + exposure/adherence + confounders)	Use as a “reference anchor” for future comparisons (personal nadir)	(6, 7, 10, 57, 80)
Maintenance monitoring	Every 6–12 months *(or with annual MRI)*	Ongoing surveillance for biological breakthrough	Stable low with small biological variability	Reproducible re-elevation (on ≥2 samples) suggests renewed injury → targeted work-up	Frequency should match clinical risk: higher-risk patients may benefit from 6-monthly; stable patients often yearly	(15, 19, 57)
Event-triggered testing	~6–12 weeks after suspected relapse/new MRI activity	Capture the peak window of injury signal after an event	Transient peak then decline if event resolves and therapy controls inflammation	If peak is unusually large/prolonged or continues to rise: consider ongoing activity, extensive lesion burden, or non-MS cause	Timing matters: sampling too early may miss the peak; too late may miss it entirely	(7, 8, 13, 80)
After switching/interruption	Baseline at switch + ~ 3 months + ~ 6 months	Distinguish carryover vs. breakthrough/rebound vs. recovery	Expected decline if new regimen suppresses inflammatory activity	Sustained rise after switch/interruption increases concern for breakthrough/rebound or inadequate exposure	Interpret dynamically; ensure consistent platform; correlate tightly with MRI timing and clinical course	(10, 19, 57, 80)

### 7.2 Patterns of sNfL response under disease-modifying therapies

Rather than differing primarily by drug class, DMTs show reproducible patterns of sNfL response that reflect the degree, speed, and durability of suppression of inflammatory neuroaxonal injury. This pattern-based approach is often more clinically useful than cataloguing individual agents, because it translates biomarker dynamics into categories that can be integrated with MRI and clinical assessment (15, 79, 80) (Table 8).

**Table 8:** Maps typical sNfL response patterns to DMT families: expected trajectories and clinical interpretation.

DMT class	Expected sNfL trajectory	Time to biological effect	Frequency of residual elevation	Main biological interpretation	Clinical implication	Key references
Platform injectables (interferons, glatiramer acetate)	Modest decline; frequent residual elevation	3–6 months	Common	Partial suppression of inflammatory axonal injury	Persistent elevation compatible with subclinical activity; reassess MRI and treatment adequacy	(7, 10)
Moderate-efficacy oral therapies (teriflunomide, fumarates)	Intermediate decline; heterogeneous trajectories	3–9 months	Moderate	Variable inflammatory control depending on baseline burden	Rising or persistently high sNfL supports closer monitoring or escalation	(7, 10, 80)
S1P receptor modulators	Rapid and sustained decline in responders	1–3 months	Uncommon	Effective suppression of inflammatory trafficking	Re-elevation raises concern for breakthrough disease or rebound	(7, 10, 80)
Anti-CD20 monoclonal antibodies	Pronounced and durable suppression	1–3 months	Rare	Near-complete control of inflammatory axonal injury	Persistent elevation suggests biological non-response or alternative pathology	(7, 10, 40, 80)
Natalizumab (including EID)	Marked suppression; stable low trajectories	1–3 months	Rare in stable patients	Potent inhibition of leukocyte CNS entry	Sudden rise warrants urgent reassessment (rebound, PML-IRIS, other injury)	(7, 10, 80, 81)
Immune reconstitution therapies (alemtuzumab, cladribine, AHSCT)	Sharp decline followed by prolonged stability	1–6 months	Low if responder	Reset of inflammatory biology	Re-elevation during drug-free intervals suggests reactivation or intercurrent cause	(44, 79, 80)
Treatment interruption/switching	Transient rise possible	Variable	Context-dependent	Rebound or immune reconstitution injury	Interpret dynamically; confirm and correlate with MRI and clinical status	(7, 10, 19, 82)

### 7.3 Partial and delayed suppression

Therapies with modest or intermediate anti-inflammatory efficacy are typically associated with gradual and incomplete reductions in sNfL, particularly in patients with high baseline inflammatory burden. In these settings, sNfL may decline relative to pre-treatment values yet remain above age-expected ranges or show intermittent low-grade elevations. This pattern is compatible with residual subclinical inflammatory activity, especially early after treatment initiation or in highly active disease.

Clinically, persistently elevated sNfL in this context should not be labeled “treatment failure” in isolation; rather, it may justify closer follow-up and targeted reassessment when supported by MRI or clinical signals.

### 7.4 Rapid and sustained normalization

Highly effective anti-inflammatory therapies are characterized by rapid and profound suppression of sNfL—often detectable within weeks to months—followed by sustained low or age-expected levels during continued disease control. This trajectory generally parallels suppression of relapses and MRI inflammatory activity and is biologically consistent with prevention of ongoing neuroaxonal injury.

Importantly, sustained low sNfL does not exclude gradual progression driven by non-inflammatory mechanisms, reinforcing the need for complementary biomarkers (e.g., sGFAP) and structural measures when disability worsens despite apparent inflammatory control.

### 7.5 Re-elevation after initial suppression: biological breakthrough signals

A reproducible rise in sNfL after prior suppression may signal renewed neuroaxonal injury. Potential explanations include breakthrough inflammatory activity, rebound phenomena, treatment interruption, suboptimal exposure/adherence, or intercurrent neurological/systemic insults. This pattern is most informative when confirmed on serial testing using the same platform and interpreted alongside symptoms, MRI timing, or potential confounders.

### 7.6 Durable suppression after immune reconstitution

Following immune reconstitution therapies, sNfL often shows a marked post-treatment decline with prolonged maintenance of low levels during drug-free intervals in responders. This pattern reflects durable suppression of inflammatory neuroaxonal injury rather than continuous pharmacodynamic exposure. Re-elevation during follow-up should prompt reassessment, but it does not by itself define loss of effect or mandate retreatment, because validated sNfL thresholds for re-dosing are not available.

### 7.7 Biological non-response and safety-relevant surges

A practical contribution of longitudinal sNfL monitoring is the detection of biological non-response, defined pragmatically as failure to show the expected on-treatment decline or confirmed re-elevation despite apparent clinical stability (15, 19). The appropriate response is structured reassessment: verify analytical consistency, repeat testing when appropriate, review adherence and treatment exposure, exclude non-MS confounders, and perform targeted MRI.

In selected safety-related contexts, marked and abrupt sNfL surges may function as a non-specific biological alarm signal. Such increases have been reported in severe inflammatory rebound, progressive multifocal leukoencephalopathy–associated immune reconstitution, and high-risk treatment sequencing, where they likely reflect extensive neuroaxonal injury rather than disease-specific pathology (81, 82). sNfL is not a screening or diagnostic test for treatment-related complications. Its value is greatest when changes are pronounced, rapid, and temporally concordant with clinical deterioration (Table 9).

**Table 9:** High-risk clinical scenarios where abrupt sNfL surges warrant urgent contextual evaluation (example contexts, key differential considerations, recommended corroboration).

Context/trigger	Typical sNfL pattern	Key differentials/pitfalls	Practical response (what to do)	Key references
New severe neurologic deficit/suspected severe relapse with high lesion burden (brain and/or spinal cord)	Marked rise relative to baseline; peaks over days–weeks	Timing vs. relapse/MRI activity; steroid exposure; assay/platform change	Urgent clinical review; expedited MRI (brain ± spine); manage relapse per standards; repeat sNfL on same platform to confirm trend	(6–8, 10, 15)
Natalizumab-treated patient with atypical symptoms or MRI concern for PML/PML-IRIS	Very high or rapidly rising; may exceed typical relapse levels; pre-diagnostic rise reported	Severe relapse; other CNS infection; IRIS after withdrawal	Immediate MRI with DWI; CSF JCV PCR as indicated; follow PML work-up pathway; do not attribute to MS relapse alone	(81, 82)
Immunosuppressed patient with systemic infection or suspected CNS infection (encephalitis/meningitis)	Potentially high, non-specific rise	Fever/inflammation; renal dysfunction; seizures; sampling timing	Urgent systemic and neurologic evaluation; consider MRI/CSF when indicated; repeat after recovery to re-establish baseline	(13, 15)
Acute cerebrovascular event (stroke/ICH/SAH) or hypoxic injury	High rise; magnitude relates to injury severity	Misattribution to MS inflammatory activity	Treat as neurologic emergency; neuroimaging/vascular work-up; interpret sNfL as non-MS injury signal	(13, 15, 24)
Head or spinal trauma (including concussion)	Abrupt rise after injury; may remain elevated for weeks	Minor trauma overlooked; recent exercise/accidents	Document trauma; avoid MS-directed escalation based on sNfL; repeat once stable and confounders resolved	(13, 24)
Status epilepticus or frequent seizures	Transient rise possible	Subclinical seizures; medication changes; intercurrent illness	Evaluate seizure etiology (EEG/MRI as appropriate); repeat sNfL after stabilization	(13)
Unexpected surge around DMT switch/withdrawal or shortly after steroids	Interpretation depends on timing; may reflect pre-treatment activity or sampling window	Washout effects; delayed lesion maturation; steroid timing	Anchor to clinical/MRI context; repeat at standardized interval on same platform; avoid switching therapy on sNfL alone	(7, 10, 15, 19)

### 7.8 Clinical take-home

In clinical practice, the central question is not whether sNfL decreases at one timepoint, but whether suppression is sustained within an appropriate reference framework. An early and durable decline supports effective suppression of inflammatory neuroaxonal injury; stable low or age-expected trajectories support ongoing inflammatory control, although they do not exclude progression; and confirmed re-elevation warrants reassessment rather than dismissal as biological variability.

Accordingly, sNfL is best framed as a pharmacodynamic and monitoring biomarker that helps contextualize uncertainty, support monitoring intensity, and guide targeted reassessment. It may inform escalation or de-escalation discussions when concordant with clinical and radiological evidence, but it should not override discordant clinical signals or be used as a stand-alone response criterion (15, 80).

## 8 sNfL across the MS continuum: from preclinical disease to established MS

Multiple sclerosis is increasingly understood as a biological continuum in which inflammatory injury and neuroaxonal damage can precede clinical manifestation by years. Within this framework, serum neurofilament light chain (sNfL) has emerged as a sensitive circulating biomarker capturing the tempo and burden of axonal injury across disease stages—from preclinical risk states to established relapsing MS. Its interpretation remains contextual: sNfL is most useful for risk enrichment and biological staging in defined at-risk populations, and should be integrated with MRI, CSF, and clinical features.

### 8.1 Preclinical MS and the prodromal phase

Large population-based biobank studies show that individuals who later develop MS have, on average, higher sNfL concentrations years before symptom onset, with divergence from matched controls detectable up to 6–10 years prior to the first demyelinating event (83, 84). These findings support the concept of a biologically active prodromal phase preceding clinical recognition of MS, accompanied by evolving patterns of healthcare utilization and neurological symptoms before overt disease onset (85).

Although these presymptomatic elevations typically overlap substantially with age-adjusted normative ranges, they reinforce the concept that neuroaxonal injury may precede clinical manifestation by several years. In this setting, sNfL is best understood as a pathobiological and risk-enrichment biomarker rather than a diagnostic test, particularly when interpreted alongside MRI findings, EBV-related markers, and other biological or clinical risk indicators (15, 56).

### 8.2 Radiologically isolated syndrome (RIS)

Radiologically isolated syndrome represents a transitional state between asymptomatic CNS pathology and clinically manifest MS. Early CSF studies demonstrated elevated neurofilament levels in RIS, indicating ongoing neuroaxonal injury despite absence of overt symptoms (86). More recent work has reinforced the concept that RIS may represent biologically active disease in a subset of individuals at higher risk of conversion, including those with inflammatory imaging features or evolving biomarker abnormalities (87, 88).

Within this framework, multimodal biomarker approaches integrating serum and CSF markers with MRI and clinical features are increasingly being explored for risk stratification and biological staging in RIS and related neuroinflammatory conditions (89, 90). Clinically, elevated age-adjusted sNfL may support closer surveillance or trial eligibility when concordant with established high-risk features, whereas normal levels do not exclude future inflammatory activity.

### 8.3 Clinically isolated syndrome (CIS)

In CIS, sNfL reflects the acute/subacute neuroaxonal injury associated with the index inflammatory event and is consistently higher than in healthy controls (91, 92). Elevated baseline sNfL is associated with increased risk of conversion to clinically definite MS, higher lesion accrual, and accelerated brain atrophy—often with disproportionate effects on grey matter (93, 94).

Across multivariable models, however, the prognostic contribution of sNfL is typically secondary to baseline MRI features and CSF oligoclonal bands. In practice, sNfL refines risk stratification when integrated with imaging and CSF data, rather than determining treatment decisions in isolation (15).

### 8.4 Early relapsing MS

Following clinical diagnosis, sNfL often peaks during early inflammatory phases and declines with effective therapy. Longitudinal trajectories spanning RIS, CIS, and early relapsing MS indicate that persistent elevation or recurrent sNfL peaks are associated with higher risk of subsequent relapses, MRI activity, and disability accumulation, whereas durable suppression is associated with more favorable outcomes (7, 10).

In early relapsing MS, the most actionable use of sNfL is trajectory monitoring: sustained low or age-expected levels support biological inflammatory control, while reproducible re-elevation—especially when temporally consistent—supports targeted reassessment (MRI review, adherence/exposure evaluation, and exclusion of confounders) (Table 10).

**Table 10:** Role and limitations of sNfL across early stages of the MS continuum.

Disease stage	Typical sNfL behavior	Clinical value	Main limitations	Key references
General population/prodromal MS	Mild elevation years before onset; overlaps with age-adjusted normative ranges	Pathobiological insight; potential risk-enrichment marker in defined at-risk populations	Not suitable for screening; low specificity and marked overlap with normal aging	(83–85)
Radiologically isolated síndrome (RIS)	Frequently elevated in individuals with higher-risk radiological or inflammatory profiles	Risk stratification; prediction of conversion	Not a standalone decision tool; interpretation remains probabilistic	(86–90)
Clinically isolated síndrome (CIS)	Markedly elevated at event or shortly after; prognostic for conversion and atrophy	Prognostic modifier when combined with MRI/OCB	Adds prognostic value when integrated with MRI/OCB, but is generally secondary to these established predictors	(91–94)
Early relapsing MS (RRMS)	Peaks with inflammatory activity; declines with treatment	Longitudinal monitoring; treatment response	Limited sensitivity for silent progression	(7, 10, 15)

## 9 Clinical interpretation of sNfL in real-world practice: trajectories and biologically informative discordances

In routine practice, sNfL is often most informative precisely in situations of uncertainty or discordance, where conventional clinical assessment or MRI provides incomplete insight into ongoing biological injury. Importantly, interpretation requires within-platform consistency, awareness of physiological confounders, and a focus on reproducible patterns rather than single outlying values (8, 10, 15, 61).

### 9.1 From isolated measurements to longitudinal trajectories

Across cohorts and disease stages, sustained increases relative to an individual baseline or nadir are consistently associated with higher short-term risk of inflammatory activity and, over longer horizons, with disability accumulation—even when absolute concentrations remain within age-expected ranges (8–10). In practice, the goal is to establish an individual reference trajectory: a baseline (preferably during clinical/MRI quiescence), an on-treatment nadir where applicable, and subsequent monitoring for meaningful deviation from that personal track.

Trajectory interpretation is strengthened when changes are confirmed on serial measurements obtained several months apart and analyzed on the same platform. Confirmed upward trajectories should prompt evaluation rather than reassurance (15, 19). Conversely, persistently low and stable trajectories are associated with a lower probability of near-term inflammatory reactivation, while still not excluding slow progression. Accordingly, sNfL functions as a dynamic sensor of recent neuroaxonal injury, whose meaning emerges primarily through patterns over time.

### 9.2 Biologically informative discordance between sNfL, MRI, and clinical status

Discordance between sNfL levels, MRI findings, and clinical status is common—and often biologically informative. At the population level, sNfL changes frequently lag focal inflammatory lesion dynamics and may peak weeks after inflammatory events, reflecting release and clearance kinetics rather than real-time activity (7, 64, 65).

Such discordance may reflect subclinical inflammatory injury, involvement of compartments less well captured by routine imaging, delayed biomarker normalization after recent activity, aging-related vulnerability, or non-MS-related confounders. Importantly, normal or low sNfL does not exclude focal inflammatory activity, particularly when lesions are anatomically restricted or sampling occurs outside the optimal temporal window. Overall, these scenarios underscore that sNfL complements—but does not replace—MRI and clinical assessment.

A particularly frequent real-world scenario is sustained sNfL elevation without relapses or overt MRI activity. Rather than being dismissed as noise, this pattern warrants structured reappraisal: confirm analytical consistency, repeat sampling on the same platform, review timing relative to recent events, exclude systemic confounders, and consider targeted imaging when appropriate (15).

## 10 Integration with GFAP and structural biomarkers

Joint interpretation with serum glial fibrillary acidic protein (sGFAP) and structural measures further refines biological meaning by separating predominantly inflammatory injury from progression-dominated biology. In broad terms, high or rising sNfL with relatively lower sGFAP is more consistent with acute/subacute inflammatory neuroaxonal injury, whereas elevated sGFAP with normal or mildly increased sNfL supports chronic astroglial activation and tissue remodelling often associated with progression, particularly in later disease stages (27, 30, 37).

Structural biomarkers help contextualize this further. Quantitative MRI and optical coherence tomography primarily capture accumulated tissue loss over years, whereas sNfL reflects recent neuroaxonal injury over weeks to months. This temporal dissociation likely contributes to the frequent partial decoupling between fluid biomarkers and established structural damage (8, 10, 95).

Clinically, a rising sNfL trajectory in the presence of limited atrophy may signal ongoing injury at a potentially modifiable stage, whereas advanced atrophy with low or stable sNfL—sometimes accompanied by higher sGFAP—may be more suggestive of a later-stage, neurodegeneration-weighted biology with less overt inflammatory activity, in which purely anti-inflammatory escalation is less likely to yield substantial benefit (27, 30, 37, 95).

### 10.1 Practical synthesis: pattern recognition over thresholds

Taken together, these observations support a contextual, multimodal approach to sNfL interpretation in which longitudinal trajectories and biologically meaningful discordances guide clinical reasoning. Rather than seeking universal numerical cut-offs, clinicians gain the most from recognizing recurring interpretive patterns—stable suppression, delayed normalization after activity, persistent elevation, and reproducible re-elevation—and responding with proportionate reassessment steps integrated with MRI and clinical evaluation (15, 19) (Table 11).

**Table 11:** Common interpretative patterns and discordant biomarker scenarios in MS.

sNfL trajectory	MRI activity	GFAP	Clinical context	Most likely biological interpretation	Practical clinical action	Key references
Rising	Active	Low–normal	Relapse or subclinical activity	Ongoing inflammatory neuroaxonal injury	Reassess disease control, repeat MRI	(6–8, 10, 15)
Rising	Inactive	Low	Clinically stable	Subclinical inflammatory activity or delayed kinetics	Closer monitoring, repeat sNfL	(7, 15, 80)
Low–stable	Active	Low	New isolated lesion	Limited focal inflammation	MRI-driven decisions	(6, 7, 80)
Low–stable	Inactive	High	Progressive phenotype	Progression-dominated biology	Focus on neuroprotection, rehab	(36, 37, 73, 74)
High stable	Inactive	High	Older patient	Age-related vulnerability and/or PIRA-related biology	Multimodal assessment	(15, 56, 73, 74)
Abrupt surge(see Table 9 for detailed differential diagnosis)	Variable	Variable	Safety context	Extensive axonal injury	Urgent reassessment	(13, 15, 24)

### 10.2 Advanced clinical scenarios where sNfL informs decision-making

In routine MS care, sNfL is most valuable in risk-sensitive scenarios characterized by uncertainty or discordance. Across these contexts, sNfL should be interpreted as a decision-support biological signal: it quantifies the recent burden of neuroaxonal injury over weeks to months, but it does not specify cause and cannot replace neurological assessment or MRI (15).

### 10.3 Clinical–radiological discordance

Discordance between sNfL trajectories, MRI activity, and clinical status is common and biologically expected because these measures operate on different time scales and capture distinct domains.

*Pattern A*: rising or persistently elevated sNfL with stable MRI and no relapses.

This pattern may reflect

- subclinical inflammatory injury below MRI detection thresholds;
- activity in less well-visualized compartments (spinal cord, cortical/subpial pathology);
- delayed normalization after recent inflammatory events;
- non–MS-related causes.

This scenario warrants structured reassessment: confirm analytical consistency, repeat sampling, review timing and confounders, and tailor imaging when appropriate (15).

*Pattern B*: MRI activity and/or relapses with low or stable sNfL. This may occur when inflammatory lesions are small or strategically located (driving symptoms with limited global axonal spillover), when sampling is suboptimal, or when treatment partially suppresses the systemic biomarker response. In such cases, management should remain anchored in integrated clinical–radiological assessment, using sNfL as complementary rather than determinative evidence (7, 96).

Across discordant situations, the confirmed trajectory relative to an individual baseline/nadir is often more informative than any single absolute value.

## 11 Treatment adaptation under uncertainty

Treatment decisions are often made under incomplete or discordant information. In this context, longitudinal sNfL can support reasoning by strengthening or weakening suspicion of ongoing neuroaxonal injury and helping prioritize follow-up and imaging when conventional measures are inconclusive (15, 19).

### 11.1 Anti-CD20 interval extension

In patients treated with anti-CD20 therapies, interval extension strategies are increasingly considered in clinically stable individuals. Longitudinal sNfL trajectories may provide a pragmatic adjunct for monitoring biological stability: persistently low and stable sNfL levels are compatible with effective suppression of inflammatory neuroaxonal injury, whereas rising trajectories may raise suspicion of residual or re-emerging activity.

However, evidence remains limited, and sNfL should be interpreted as supportive information within an integrated clinical and radiological assessment, particularly during extended dosing (10, 15, 97).

### 11.2 Unexpected biomarker trajectories under treatment

Unexpected changes in sNfL in clinically stable patients or in those with discordant MRI findings represent situations of biological uncertainty rather than immediate treatment failure. A reproducible increase should prompt structured reassessment—confirming analytical consistency, reviewing timing and potential confounders, and considering targeted MRI—rather than automatic treatment escalation (15, 19).

Under natalizumab treatment, particularly in higher-risk monitoring contexts such as treatment interruption or extended-interval dosing strategies, the value of sNfL lies primarily in identifying unexpected biological changes. A marked and unexplained rise in sNfL should prompt urgent reassessment and MRI-based work-up, including consideration of progressive multifocal leukoencephalopathy (PML) in the appropriate clinical context (81).

### 11.3 Safety-critical red flags: PML, IRIS, and severe CNS injury

Marked or rapidly rising sNfL levels represent a shift from interpretative uncertainty to urgent evaluation. These patterns reflect substantial acute neuroaxonal injury and may occur in severe inflammatory rebound, opportunistic infections, progressive multifocal leukoencephalopathy (PML), PML-associated immune reconstitution inflammatory syndrome (IRIS), and other acute CNS insults such as stroke or trauma (81, 82, 98).

Although non-specific, these elevations are typically more pronounced than those seen in routine MS activity and should prompt immediate clinical and radiological assessment. Case-based evidence suggests that in natalizumab-associated PML, sNfL levels may be substantially higher than in typical relapses and, in some cases, may rise before overt diagnosis. Similar patterns have been described in PML-IRIS and other opportunistic complications under DMTs (81, 82, 98). Table 9 summarizes representative high-risk scenarios in which abrupt sNfL surges warrant urgent contextual evaluation.

## 12 Special populations

### 12.1 Pregnancy, postpartum, and lactation

Pregnancy and postpartum combine true changes in relapse biology with physiological modifiers and practical constraints on MRI (particularly gadolinium). In this setting, sNfL interpretation is particularly challenging.

Prospective data suggest that sNfL in early pregnancy may be influenced by physiological changes (including hemodilution) and may not reliably reflect inflammatory activity, whereas postpartum increases appear to align more closely with relapse risk in some cohorts (99).

During breastfeeding, transient maternal fluctuations may occur without overt clinical activity, and NfL detected in breast milk currently lacks clinical interpretability (100).

In selected treatment contexts, pharmacokinetic and pharmacodynamic studies during pregnancy and lactation further support the role of sNfL as a complementary monitoring biomarker, although interpretation remains highly context-dependent (101).

Practically, sNfL may support surveillance when MRI timing is constrained, but attribution to MS activity should rely on confirmed trends and supporting clinical or radiological evidence (15, 99–101).

### 12.2 Pediatric-onset MS

In pediatric multiple sclerosis, the high inflammatory burden and frequent subclinical activity make sNfL a sensitive marker of disease activity. However, interpretation requires strict age-adjusted frameworks.

Pediatric cohorts consistently demonstrate elevated sNfL levels in untreated disease and reductions following effective therapy, broadly paralleling inflammatory activity and treatment response (102, 103).

Given substantial age-related variability across childhood and adolescence, percentile- or Z-score–based approaches are preferable to fixed absolute cut-offs (56, 69) (Table 12).

**Table 12:** The current levels of evidence supporting different clinical applications of sNfL and related biomarkers in MS.

Intended clinical use	Clinical validity	Clinical utility	Implementation take-home	Key caveats	Key references
Detecting recent inflammatory disease activity (relapse and/or new/enlarging T2 or Gd + lesions) and quantifying biological impact	High	Low	Adjunct to MRI/clinical assessment; interpret trajectories and age-adjusted frameworks (percentiles/Z-scores when available).	Non-specific; timing matters; MRI remains primary for localization/acute decisions.	(6–8, 10, 15)
Individual prognostication of near-term risk of clinical/MRI disease activity (risk stratification using age-adjusted percentiles/Z-scores)	High	Low	May refine monitoring intensity (earlier MRI/repeat biomarker) in uncertain cases; avoid deterministic conclusions.	Predictive ≠ prescriptive; biomarker-guided outcome benefit not yet proven.	(8, 10, 56, 57)
Monitoring treatment response and comparative biological effects across DMT classes (on-treatment suppression vs. persistent elevation)	High	Low–Emerging	Supports interpretation of response alongside relapses/disability/MRI; consider repeat to confirm durable suppression.	Baseline strongly influences on-treatment values; avoid cross-platform comparisons.	(6, 7, 10, 57, 80, 101)
Detecting possible ‘silent’/subclinical activity in apparently stable patients	Moderate	Low	If confirmed upward trend: verify confounders → repeat with standardized timing → consider earlier MRI (incl. Spinal cord when relevant).	Intermediate values common; false positives from intercurrent illness/trauma.	(15, 19, 61)
De-escalation/discontinuation surveillance (early warning for reactivation)	Low–Moderate	Insufficient	If used, embed in structured follow-up; confirm rises with repeat testing and integrate with MRI.	Limited prospective evidence; thresholds and schedules not validated.	(15, 19, 97)
Safety triage in natalizumab-treated patients: supporting differentiation of PML vs. relapse and/or early signal of PML	Moderate	Low–Moderate (adjunct)	Alarm signal prompting urgent evaluation when suspicion exists (MRI/CSF/JCV work-up); not stand-alone screening.	Non-specific; imperfect sensitivity/specificity; interpret within full PML-risk context.	(81, 82, 98)
Progression/PIRA biology (sNfL) and neurodegeneration monitoring	Low	Insufficient	Avoid using low sNfL to rule out progression; consider complementary metrics (quantitative MRI/atrophy/spinal cord metrics, OCT).	sNfL is more inflammation-sensitive than progression-specific.	(36, 37, 73, 74)
Progression risk stratification (sGFAP), particularly in non-active progressive MS and in combination with low NfL	Moderate–High	Low–Emerging	Consider adding sGFAP when progression is a major concern; supports prognosis and monitoring strategy (not sole trigger).	Assay harmonization/normative frameworks evolving; prospective utility validation needed.	(36, 37, 73, 74, 76)
Pregnancy/postpartum monitoring (risk context, especially postpartum)	Low–Moderate	Insufficient	If measured, interpret cautiously with standardized timing and context; avoid high-stakes decisions based on isolated values.	Physiological/systemic confounding; limited prospective MS-specific data.	(99, 100)
Pediatric-onset MS	Moderate	Emerging	Use age-adjusted percentiles or Z-scores rather than fixed cut-offs	Strong age dependency limits interpretation of absolute values	(56, 69, 102, 103)

Clinical validity reflects association and prognostic performance, whereas clinical utility reflects evidence that biomarker-guided actions improve outcomes. Evidence level key: High = consistent findings across multiple independent cohorts/meta-analyses; Moderate = multiple studies with heterogeneity/limitations; Low = limited/inconsistent data; Insufficient = not adequate to support routine use or outcome-driven decisions.

### 12.3 De-escalation and deprescribing

De-escalation represents a biologically relevant use case for sNfL, as the main risk is inflammatory reactivation after reduced treatment exposure. Persistently low or stable trajectories are compatible with absence of major new inflammatory injury when supported by clinical and MRI stability. Conversely, a confirmed rise should prompt reassessment for renewed inflammatory activity or alternative causes and may support re-intensification when corroborated (15, 19) (Supplementary Table S1).

## 13 Clinical implementation and consensus-based decision frameworks (CMSC-informed)

In the absence of regulatory, ‘label-like’ action thresholds, clinical implementation of serum neurofilament light chain (sNfL) has been guided by expert consensus statements and best-practice recommendations. CMSC-led guidance, Delphi-based consensus frameworks and national recommendations provide a pragmatic foundation for integrating sNfL into routine clinical care (15, 19, 20, 104).

Across these frameworks, sNfL is conceptualized as a biomarker of recent neuroaxonal injury that supports clinical reasoning and risk stratification within an integrated clinical-radiological context. A practical implementation framework aligned with these principles is illustrated in Figure 1.

A practical implementation framework aligned with these principles is illustrated in Figure 1.

### 13.1 Core CMSC principles that enable safe clinical use

International and national consensus frameworks converge on a limited set of practical principles for routine use (15, 19, 20, 104):

- Interpretation should prioritize longitudinal change over isolated values;
- Results should be contextualized using age-adjusted normative frameworks;
- sNfL should be integrated with clinical and MRI findings, particularly in ambiguous scenarios;
- Rigid universal cut-offs should be avoided in favor of contextual, pattern-based interpretation

### 13.2 Practical preconditions for interpretation (reducing false signals)

Reliable interpretation requires attention to key pre-analytical and clinical factors. Longitudinal follow-up should ideally use the same analytical platform, as cross-platform differences may mimic biological change. Measurements should be interpreted in clinical context, considering intercurrent conditions that may influence sNfL levels. Timing relative to relapses, MRI activity, and treatment changes is also critical, as sNfL reflects recent injury over a delayed time window. Common pitfalls and mitigation strategies are summarized in Box 1.

### 13.3 A practical implementation mindset

In clinical practice, sNfL is best interpreted as part of a structured clinical question rather than a fixed numerical trigger. Key considerations include:

- Whether there is a meaningful change relative to the patient’s baseline or expected range;
- The most plausible explanation for that change (e.g., inflammatory activity, treatment response, delayed normalization, or alternative causes);
- And which additional data may reduce uncertainty (e.g., repeat measurement, targeted MRI, closer monitoring, or complementary biomarkers such as GFAP).

This approach reflects the intent of current consensus frameworks, which position sNfL as a tool to reduce clinical uncertainty and support proportionate decision-making, rather than as a prescriptive threshold-based marker (15, 20, 104).

## 14 Harmonizing guidance: what is established vs. what is not

Multiple guidance initiatives—including CMSC-aligned recommendations and modified Delphi frameworks—propose scenario-based pathways for clinical use (e.g., escalation, switching, or de-escalation). These frameworks provide valuable implementation support but should be interpreted as consensus-derived approaches rather than prospectively validated decision rules (15, 19, 20, 104).

For clinical application, it is essential to distinguish between:

- Established principles, supported by convergent evidence: prioritization of longitudinal interpretation, use of age-adjusted reference frameworks, and integration with clinical and MRI context.
- Unresolved areas, including biomarker-guided treatment thresholds, fixed monitoring schedules, and algorithm-driven decisions, which remain hypothesis-generating and require prospective validation (15, 20).

### 14.1 From clinical validity to clinical utility: why pragmatic trials are the missing link

Current evidence for sNfL demonstrates analytical and clinical validity, but clinical utility remains the key gap—specifically, whether biomarker-informed decisions improve patient outcomes.

Addressing this gap requires pragmatic randomized trials embedded in routine care settings, an approach increasingly recognized in biomarker implementation research (105). The MultiSCRIPT study exemplifies this strategy by evaluating structured sNfL-guided management compared with standard monitoring (106).

Strategy trials such as TREAT-MS and DELIVER-MS further illustrate how pragmatic designs can address clinically relevant questions when conventional randomized trials are not feasible (107, 108).

At present, sNfL should be considered a decision-support biomarker under active evaluation rather than an outcome-validated decision-making tool (Supplementary Table S2).

### 14.2 Extending biological coverage: the role of sGFAP

Serum glial fibrillary acidic protein (sGFAP) provides complementary biological information by reflecting astroglial activation and chronic tissue stress. Its most relevant clinical role lies in scenarios of disability worsening with limited inflammatory activity, where it may support a progression-dominant interpretation when considered alongside sNfL.

However, standardization, normative frameworks, and prospective validation remain incomplete. Accordingly, sGFAP should be considered an adjunct biomarker that may reduce uncertainty rather than a prescriptive decision-making tool.

### 14.3 Limitations that define appropriate use

sNfL has intrinsic limitations that must be acknowledged to prevent overinterpretation. It is non-specific, reflects recent injury more than slow progression, and remains analytically context-dependent, with limited comparability across platforms. Most importantly, no sNfL threshold has been prospectively validated as a trigger for treatment modification in routine care (15, 20).

### 14.4 Knowledge gaps and future directions: from association to action

Future work should prioritize clinical utility over further accumulation of associative data. Key priorities include:

- Harmonization of assays and development of robust normative frameworks.
- Validation in underrepresented and clinically complex populations.
- Integration of multimodal biomarkers (sNfL, sGFAP, imaging).
- Development of trajectory-based monitoring approaches.

Emerging machine-learning approaches integrating fluid biomarkers and imaging data may enable more precise disease stratification, although these strategies remain in early stages of validation (109). Implementation must also remain scalable and equitable to avoid widening healthcare disparities.

## 15 Conclusion

Serum neurofilament light chain (sNfL) has consolidated its role as a robust and extensively validated blood-based biomarker of neuroaxonal injury in multiple sclerosis (8–10, 15). Its clinical value lies in providing a reproducible signal of recent axonal injury that, when interpreted longitudinally and in context, can meaningfully support clinical reasoning—particularly in situations of uncertainty, apparent stability, or clinical–radiological discordance.

Its principal strengths include sensitivity to inflammation-associated injury, its role as a pharmacodynamic marker under treatment, and its ability to refine short-term biological risk when interpreted over time. At the same time, these strengths define its limitations. sNfL is not disease-specific, is influenced by physiological and systemic factors, and has limited sensitivity for non-inflammatory progression, particularly in later disease stages (13, 69).

Accordingly, its role in clinical practice is not to dictate decisions, but to reduce uncertainty and guide proportionate next steps—whether reinforcing current management, prompting closer monitoring, or supporting targeted reassessment. In this sense, sNfL should be regarded as a decision-support biomarker rather than an outcome-validated decision-making tool.

The next phase of translation will depend on demonstrating clinical utility. As analytical harmonization advances and pragmatic trials clarify the impact of biomarker-informed strategies on patient outcomes, integration of sNfL with complementary biomarkers—particularly GFAP—and quantitative imaging may enable a more biologically informed and individualized model of multiple sclerosis care (37, 109).

Ultimately, the value of sNfL will be determined not by its ability to measure injury, but by its capacity to improve decisions and outcomes in real-world clinical practice.

## References

[1] Compston A Coles A. Multiple sclerosis. Lancet. (2008) 372:1502–17. doi: 10.1016/S0140-6736(08)61620-7, 18970977

[2] Reich DS Lucchinetti CF Calabresi PA. Multiple Sclerosis. N Engl J Med. (2018) 378:169–80. doi: 10.1056/NEJMra1401483, 29320652 PMC6942519

[3] Montalban X Piasecka-Stryczynska K Kuhle J Benkert P Arnold DL Weber MS . Efficacy and safety results after >3.5 years of treatment with the Bruton's tyrosine kinase inhibitor evobrutinib in relapsing multiple sclerosis: long-term follow-up of a phase II randomised clinical trial with a cerebrospinal fluid sub-study. Mult Scler. (2024) 30:558–70. doi: 10.1177/13524585241234783, 38436271 PMC11080380

[4] Saidha S Sotirchos ES Oh J Syc SB Seigo MA Shiee N . Relationships between retinal axonal and neuronal measures and global central nervous system pathology in multiple sclerosis. JAMA Neurol. (2013) 70:34–43. doi: 10.1001/jamaneurol.2013.573, 23318513 PMC4030557

[5] Kuhle J Barro C Andreasson U Derfuss T Lindberg R Sandelius Å. Comparison of three analytical platforms for quantification of the neurofilament light chain in blood samples: ELISA, electrochemiluminescence immunoassay and Simoa. Clin Chem Lab Med. (2016) 54:1655–61. doi: 10.1515/cclm-2015-1195, 27071153

[6] Disanto G Barro C Benkert P Naegelin Y Schädelin S Giardiello A . Serum Neurofilament light: a biomarker of neuronal damage in multiple sclerosis. Ann Neurol. (2017) 81:857–70. doi: 10.1002/ana.24954, 28512753 PMC5519945

[7] Novakova L Zetterberg H Sundström P Axelsson M Khademi M Gunnarsson M . Monitoring disease activity in multiple sclerosis using serum neurofilament light protein. Neurology. (2017) 89:2230–7. doi: 10.1212/WNL.0000000000004683, 29079686 PMC5705244

[8] Barro C Benkert P Disanto G Tsagkas C Amann M Naegelin Y . Serum neurofilament as a predictor of disease worsening and brain and spinal cord atrophy in multiple sclerosis. Brain. (2018) 141:2382–91. doi: 10.1093/brain/awy154, 29860296

[9] Cantó E Barro C Zhao C Caillier SJ Michalak Z Bove R . Association between serum Neurofilament light chain levels and long-term disease course among patients with multiple sclerosis followed up for 12 years. JAMA Neurol. (2019) 76:1359–66. doi: 10.1001/jamaneurol.2019.2137, 31403661 PMC6692664

[10] Benkert P Meier S Schaedelin S Manouchehrinia A Yaldizli Ö Maceski A . Serum neurofilament light chain for individual prognostication of disease activity in people with multiple sclerosis: a retrospective modelling and validation study. Lancet Neurol. (2022) 21:246–57. doi: 10.1016/S1474-4422(22)00009-6, 35182510

[11] Benkert P Maleska Maceski A Schaedelin S Oechtering J Zadic A Vilchez Gomez JF . Serum glial fibrillary acidic protein and Neurofilament light chain levels reflect different mechanisms of disease progression under B-cell depleting treatment in multiple sclerosis. Ann Neurol. (2024) 97:104–15. doi: 10.1002/ana.27096, 39411917 PMC11683165

[12] Barro C Healy BC Liu Y Saxena S Paul A Polgar-Turcsanyi M . Serum GFAP and NfL levels differentiate subsequent progression and disease activity in patients with progressive multiple sclerosis. Neurol Neuroimmunol Neuroinflamm. (2022) 10:e200052. doi: 10.1212/NXI.0000000000200052 36376097 PMC9749933

[13] Gaetani L Blennow K Calabresi P Di Filippo M Parnetti L Zetterberg H. Neurofilament light chain as a biomarker in neurological disorders. J Neurol Neurosurg Psychiatry. (2019) 90:870–81. doi: 10.1136/jnnp-2018-320106, 30967444

[14] Andreasson U Gobom J Delatour V Auclair G Noam Y Lee S . Assessing the commutability of candidate reference materials for the harmonization of neurofilament light measurements in blood. Clin Chem Lab Med. (2023) 61:1245–54. doi: 10.1515/cclm-2022-1181, 36709509

[15] Freedman MS Gnanapavan S Booth RA Calabresi PA Khalil M Kuhle J . Guidance for use of neurofilament light chain as a cerebrospinal fluid and blood biomarker in multiple sclerosis management. EBioMedicine. (2024) 101:104970. doi: 10.1016/j.ebiom.2024.104970, 38354532 PMC10875256

[16] Freedman MS Abdelhak A Bhutani MK Freeman J Gnanapavan S Hussain S . The role of serum neurofilament light (sNfL) as a biomarker in multiple sclerosis: insights from a systematic review. J Neurol. (2025) 272:400. doi: 10.1007/s00415-025-13093-1, 40372550 PMC12081536

[17] Gaetani L Schoonheim MM. Serum neurofilament light chain predicts cognitive worsening in secondary progressive multiple sclerosis better than brain MRI measures. Mult Scler. (2022) 28:1831–3. doi: 10.1177/13524585221122916, 36124836 PMC9493404

[18] Freedman MS Gnanapavan S. Letter to the editor: consensus statement on Neurofilament proteins in multiple sclerosis under development by consortium of multiple sclerosis centers (CMSC) expert panel. Int J MS Care. (2020) 22:294. doi: 10.7224/1537-2073.2020-140, 33424486 PMC7780702

[19] Yaldizli Ö Benkert P Achtnichts L Bar-Or A Bohner-Lang V Bridel C . Personalized treatment decision algorithms for the clinical application of serum neurofilament light chain in multiple sclerosis: a modified Delphi study. Mult Scler. (2025) 31:932–43. doi: 10.1177/13524585251335466, 40296363 PMC12228887

[20] Consortium of Multiple Sclerosis Centers. (2024). Best Practices Guideline for the Use of Neurofilament Light Chain in Multiple Sclerosis Care. Available online at: https://www.mscare.org/best-practices-guideline-neurofilament/ (Accessed January 28, 2026)

[21] Yusuf FLA Karim ME Sutherland JM Zhu F Zhao Y Marrie RA . Heterogeneity in health care pathways preceding the classical recognition of adult-onset multiple sclerosis: a multichannel state sequence analysis. Mult Scler Relat Disord. (2025) 102:106595. doi: 10.1016/j.msard.2025.106595, 40651073

[22] Yuan A Rao MV Veeranna Nixon RA. Neurofilaments and neurofilament proteins in health and disease. Cold Spring Harb Perspect Biol. (2017) 9:a018309. doi: 10.1101/cshperspect.a018309 28373358 PMC5378049

[23] Ding EA Kumar S. Neurofilament biophysics: from structure to biomechanics. Mol Biol Cell. (2024) 35:re1. doi: 10.1091/mbc.E23-11-0438, 38598299 PMC11151108

[24] Khalil M Teunissen CE Otto M Piehl F Sormani MP Gattringer T . Neurofilaments as biomarkers in neurological disorders. Nat Rev Neurol. (2018) 14:577–89. doi: 10.1038/s41582-018-0058-z, 30171200

[25] Daponte A Koros C Skarlis C Siozios D Rentzos M Papageorgiou SG . Neurofilament biomarkers in neurology: from Neuroinflammation to neurodegeneration, bridging established and novel analytical advances with clinical practice. Int J Mol Sci. (2025) 26:9739. doi: 10.3390/ijms26199739, 41097004 PMC12525295

[26] Siller N Kuhle J Muthuraman M Barro C Uphaus T Groppa S . Serum neurofilament light chain is a biomarker of acute and chronic neuronal damage in early multiple sclerosis. Mult Scler. (2019) 25:678–86. doi: 10.1177/1352458518765666, 29542376

[27] Chitnis T Magliozzi R Abdelhak A Kuhle J Leppert D Bielekova B. Blood and CSF biomarkers for multiple sclerosis: emerging clinical applications. Lancet Neurol. (2025) 24:1066–78. doi: 10.1016/S1474-4422(25)00249-2, 41015047

[28] Chitnis T Qureshi F Gehman VM Becich M Bove R Cree BAC . Inflammatory and neurodegenerative serum protein biomarkers increase sensitivity to detect clinical and radiographic disease activity in multiple sclerosis. Nat Commun. (2024) 15:4297. doi: 10.1038/s41467-024-48602-9, 38769309 PMC11106245

[29] Srpova B Uher T Hrnciarova T Barro C Andelova M Michalak Z . Serum neurofilament light chain reflects inflammation-driven neurodegeneration and predicts delayed brain volume loss in early stage of multiple sclerosis. Mult Scler. (2021) 27:52–60. doi: 10.1177/1352458519901272, 31961243

[30] Bittner S Oh J Havrdová EK Tintoré M Zipp F. The potential of serum neurofilament as biomarker for multiple sclerosis. Brain. (2021) 144:2954–63. doi: 10.1093/brain/awab241, 34180982 PMC8634125

[31] Paul A Comabella M Gandhi R. Biomarkers in multiple sclerosis. Cold Spring Harb Perspect Med. (2019) 9:a029058. doi: 10.1101/cshperspect.a029058, 29500303 PMC6396336

[32] Potokar M Morita M Wiche G Jorgačevski J. The diversity of intermediate filaments in astrocytes. Cells. (2020) 9:1604. doi: 10.3390/cells9071604, 32630739 PMC7408014

[33] Dong Y Benveniste EN. Immune function of astrocytes. Glia. (2001) 36:180–90. doi: 10.1002/glia.1107, 11596126

[34] Li D Liu X Liu T Liu H Tong L Jia S . Neurochemical regulation of the expression and function of glial fibrillary acidic protein in astrocytes. Glia. (2020) 68:878–97. doi: 10.1002/glia.23734, 31626364

[35] Teunissen CE Verberk IMW Thijssen EH Vermunt L Hansson O Zetterberg H . Blood-based biomarkers for Alzheimer's disease: towards clinical implementation. Lancet Neurol. (2022) 21:66–77. doi: 10.1016/S1474-4422(21)00361-6, 34838239

[36] Ayrignac X Le Bars E Duflos C Hirtz C Maleska Maceski A Carra-Dallière C . Serum GFAP in multiple sclerosis: correlation with disease type and MRI markers of disease severity. Sci Rep. (2020) 10:10923. doi: 10.1038/s41598-020-67934-2, 32616916 PMC7331703

[37] Meier S Willemse EA Schaedelin S. Serum glial fibrillary acidic protein compared with Neurofilament light chain as a biomarker for disease progression in multiple sclerosis. JAMA Neurol. (2023) 80:287–97. doi: 10.1001/jamaneurol.2022.5250, 36745446 PMC10011932

[38] Teunissen CE Khalil M. Neurofilaments as biomarkers in multiple sclerosis. Mult Scler. (2012) 18:552–6. doi: 10.1177/1352458512443092, 22492131

[39] Hemond CC Bakshi R. Magnetic resonance imaging in multiple sclerosis. Cold Spring Harb Perspect Med. (2018) 8:a028969. doi: 10.1101/cshperspect.a028969, 29358319 PMC5932576

[40] Rocca MA Preziosa P Filippi M. Advances in neuroimaging of multiple sclerosis. Curr Opin Neurol. (2025) 38:205–16. doi: 10.1097/WCO.0000000000001360, 40104925

[41] Tommasin S Giannì C De Giglio L Pantano P. Neuroimaging techniques to assess inflammation in multiple sclerosis. Neuroscience. (2019) 403:4–16. doi: 10.1016/j.neuroscience.2017.07.055, 28764938

[42] Zivadinov R Bakshi R. Role of MRI in multiple sclerosis II: brain and spinal cord atrophy. Front Biosci. (2004) 9:647–64. doi: 10.2741/1262, 14766398

[43] Sastre-Garriga J Pareto D Rovira À. Brain atrophy in multiple sclerosis: clinical relevance and technical aspects. Neuroimaging Clin N Am. (2017) 27:289–300. doi: 10.1016/j.nic.2017.01.002, 28391787

[44] Bakshi R Healy BC Dupuy SL Kirkish G Khalid F Gundel T . Brain MRI predicts worsening multiple sclerosis disability over 5 years in the SUMMIT study. J Neuroimaging. (2020) 30:212–8. doi: 10.1111/jon.12688, 31994814 PMC7194808

[45] Bostan M Pîrvulescu R Tiu C Bujor I Popa-Cherecheanu A. OCT and OCT-A biomarkers in multiple sclerosis – review. Rom J Ophthalmol. (2023) 67:107–10. doi: 10.22336/rjo.2023.20, 37522023 PMC10385714

[46] Yang J Hamade M Wu Q Wang Q Axtell R Giri S . Current and future biomarkers in multiple sclerosis. Int J Mol Sci. (2022) 23:5877. doi: 10.3390/ijms23115877, 35682558 PMC9180348

[47] Rissin DM Kan CW Campbell TG Howes SC Fournier DR Song L . Single-molecule enzyme-linked immunosorbent assay detects serum proteins at subfemtomolar concentrations. Nat Biotechnol. (2010) 28:595–9. doi: 10.1038/nbt.1641, 20495550 PMC2919230

[48] Wilson DH Rissin DM Kan CW Fournier DR Piech T Campbell TG . The Simoa HD-1 analyzer: a novel fully automated digital immunoassay analyzer with single-molecule sensitivity and multiplexing. SLAS Technol. (2016) 21:533–47. doi: 10.1177/2211068215589580, 26077162

[49] Lee S Plavina T Singh CM Xiong K Qiu X Rudick RA . Development of a highly sensitive Neurofilament light chain assay on an automated immunoassay platform. Front Neurol. (2022) 13:935382. doi: 10.3389/fneur.2022.935382, 35959400 PMC9359312

[50] Wilson D Chan D Chang L Mathis R Verberk I Montalban X . Development and multi-center validation of a fully automated digital immunoassay for neurofilament light chain: toward a clinical blood test for neuronal injury. Clin Chem Lab Med. (2023) 62:322–31. doi: 10.1515/cclm-2023-0518 37702323

[51] Coppens S. Neurofilament-light, a promising biomarker: analytical, metrological and clinical challenges. Int J Mol Sci. (2023) 24:11624. doi: 10.3390/ijms241411624, 37511382 PMC10380627

[52] Zondra Revendova K Schaffartzikova T Zeman D Hradilek P Kusnierova P. Comparison of Simoa, high sensitivity ELISA, and CLIA for serum neurofilament light chain quantification in multiple sclerosis. Sci Rep. (2025) 15:41871. doi: 10.1038/s41598-025-25926-0, 41290980 PMC12647869

[53] Dargvainiene J Torge A Wandinger KP Junker R Markewitz R Benkert P . Comparative analysis of three platforms for serum NfL quantification in healthy controls and MS patients. Clin Chem Lab Med. (2025) 64:880–8. doi: 10.1515/cclm-2025-1476, 41405952

[54] Mondesert E Delaby C De La Cruz E Kuhle J Benkert P Pradeilles N . Comparative performances of 4 serum NfL assays, pTau181, and GFAP in patients with amyotrophic lateral sclerosis. Neurology. (2025) 104:e213400. doi: 10.1212/WNL.0000000000213400, 40009787 PMC11863781

[55] Sheth U Harrison R Ferber K Rosenbaugh EG Bevis A Khillan R . Measuring neurofilament light in human plasma and cerebrospinal fluid: a comparison of five analytical immunoassays. Clin Chem Lab Med. (2025) 64:410–20. doi: 10.1515/cclm-2025-0610 40958756

[56] Simrén J Andreasson U Gobom J Suarez Calvet M Borroni B Gillberg C . Establishment of reference values for plasma neurofilament light based on healthy individuals aged 5–90 years. Brain Commun. (2022) 4:fcac174. doi: 10.1093/braincomms/fcac174, 35865350 PMC9297091

[57] Einsiedler M Maceski AM Sandgren S Oechtering J Schaedelin S Hofer L . Serum Neurofilament light chain in multiple sclerosis: superiority of age- and BMI-corrected Z scores/percentiles over absolute cutoff values for prediction of treatment response. Ann Clin Transl Neurol. (2025) 12:2214–25. doi: 10.1002/acn3.70149, 40755082 PMC12623839

[58] Bornhorst JA Figdore D Campbell MR Pazdernik VK Mielke MM Petersen RC . Plasma neurofilament light chain (NfL) reference interval determination in an age-stratified cognitively unimpaired cohort. Clin Chim Acta. (2022) 535:153–6. doi: 10.1016/j.cca.2022.08.017, 36041549

[59] Virata MCA Catahay JA Lippi G Henry BM. Neurofilament light chain: a biomarker at the crossroads of clarity and confusion for gene-directed therapies. Neurodegener Dis Manag. (2024) 14:227–39. doi: 10.1080/17582024.2024.2421738, 39545606 PMC11703492

[60] Koini M Pirpamer L Hofer E Buchmann A Pinter D Ropele S . Factors influencing serum neurofilament light chain levels in normal aging. Aging (Albany NY). (2021) 13:25729–44. doi: 10.18632/aging.203799 34923481 PMC8751593

[61] La Civita E Nicolella V Fiorenza M Cosimato V Castaldo G Morra VB . Advancing clinical use of neurofilament light chain: translational insights from research to routine practice. Biomark Insights. (2025) 20:11772719251364018. doi: 10.1177/11772719251364018, 41180593 PMC12575937

[62] Akamine S Marutani N Kanayama D Gotoh S Maruyama R Yanagida K . Renal function is associated with blood neurofilament light chain level in older adults. Sci Rep. (2020) 10:20350. doi: 10.1038/s41598-020-76990-7, 33230211 PMC7683708

[63] Thebault S Reaume M Marrie RA Marriott JJ Furlan R Laroni A . High or increasing serum NfL is predictive of impending multiple sclerosis relapses. Mult Scler Relat Disord. (2022) 59:103535. doi: 10.1016/j.msard.2022.103535, 35078125

[64] Rosso M Gonzalez CT Healy BC Saxena S Paul A Bjornevik K . Temporal association of sNfL and gad-enhancing lesions in multiple sclerosis. Ann Clin Transl Neurol. (2020) 7:945–55. doi: 10.1002/acn3.51060, 32452160 PMC7318095

[65] Fox RJ Cree BAC de Sèze J Gold R Hartung HP Jeffery D . Temporal relationship between serum Neurofilament light chain and radiologic disease activity in patients with multiple sclerosis. Neurology. (2024) 102:e209357. doi: 10.1212/WNL.0000000000209357, 38648580 PMC11175646

[66] Uher T Schaedelin S Srpova B Barro C Bergsland N Dwyer M . Monitoring of radiologic disease activity by serum neurofilaments in MS. Neurol Neuroimmunol Neuroinflamm. (2020) 7:e714. doi: 10.1212/NXI.0000000000000714, 32273481 PMC7176248

[67] Uher T McComb M Galkin S Srpova B Oechtering J Barro C . Neurofilament levels are associated with blood-brain barrier integrity, lymphocyte extravasation, and risk factors following the first demyelinating event in multiple sclerosis. Mult Scler. (2021) 27:220–31. doi: 10.1177/1352458520912379, 32255388

[68] Sotirchos ES Hu C Smith MD Lord HN DuVal AL Arrambide G . Agreement between published reference resources for neurofilament light chain levels in people with multiple sclerosis. Neurology. (2023) 101:e2448–53. doi: 10.1212/WNL.0000000000207957, 37816633 PMC10752633

[69] Abdelhak A Benkert P Schaedelin S Boscardin WJ Cordano C Oechtering J . Neurofilament light chain elevation and disability progression in multiple sclerosis. JAMA Neurol. (2023) 80:1317–25. doi: 10.1001/jamaneurol.2023.3997, 37930670 PMC10628837

[70] Abdelhak A Antweiler K Kowarik MC Senel M Havla J Zettl UK . Serum glial fibrillary acidic protein and disability progression in progressive multiple sclerosis. Ann Clin Transl Neurol. (2024) 11:477–85. doi: 10.1002/acn3.51969, 38111972 PMC10863922

[71] Manouchehrinia A Stridh P Khademi M Leppert D Barro C Michalak Z . Plasma neurofilament light levels are associated with risk of disability in multiple sclerosis. Neurology. (2020) 94:e2457–67. doi: 10.1212/WNL.0000000000009571, 32434867 PMC7455371

[72] Thebault S Abdoli M Fereshtehnejad SM Tessier D Tabard-Cossa V Freedman MS. Serum neurofilament light chain predicts long term clinical outcomes in multiple sclerosis. Sci Rep. (2020) 10:10381. doi: 10.1038/s41598-020-67504-6, 32587320 PMC7316736

[73] Maceski AM Benkert P Einsiedler M Schaedelin S Oechtering J Melie-Garcia L . GFAP and NfL as predictors of disease progression and relapse activity in fingolimod-treated multiple sclerosis. Brain. (2025):awaf433. doi: 10.1093/brain/awaf433, 41237262 PMC13337245

[74] Comabella M Sastre-Garriga J Carbonell-Mirabent P Fissolo N Tur C Malhotra S . Serum neurofilament light chain levels predict long-term disability progression in patients with progressive multiple sclerosis. J Neurol Neurosurg Psychiatry. (2022) 93:732–40. doi: 10.1136/jnnp-2022-329020, 35487685

[75] Akgün K Kretschmann N Haase R Proschmann U Kitzler HH Reichmann H . Profiling individual clinical responses by high-frequency serum neurofilament assessment in MS. Neurol Neuroimmunol Neuroinflamm. (2019) 6:e555. doi: 10.1212/NXI.0000000000000555, 31119188 PMC6501638

[76] Thebault S Fereshtehnejad SM Bergman HP Breville G Abdoli M Booth RA . The combination of CSF neurofilament light chain and glial fibrillary acidic protein improves the prediction of long-term confirmed disability worsening in multiple sclerosis. Sci Rep. (2024) 14:29135. doi: 10.1038/s41598-024-75290-8, 39587121 PMC11589836

[77] Monreal E Fernández-Velasco JI Álvarez-Lafuente R Sainz de la Maza S García-Sánchez MI Llufriu S . Serum biomarkers at disease onset for personalized therapy in multiple sclerosis. Brain. (2024) 147:4084–93. doi: 10.1093/brain/awae260, 39101570

[78] Monreal E Fernández-Velasco JI García-Sánchez MI. Association of Serum Neurofilament Light Chain Levels at disease onset with disability worsening in patients with a first demyelinating multiple sclerosis event not treated with high-efficacy drugs. JAMA Neurol. (2023) 80:397–403. doi: 10.1001/jamaneurol.2023.0010, 36848127 PMC9972238

[79] Wiendl H Barkhof F Montalban X Achiron A Derfuss T Chan A . Blood biomarker dynamics in people with relapsing multiple sclerosis treated with cladribine tablets: results of the 2-year MAGNIFY-MS study. Front Immunol. (2025) 16:1512189. doi: 10.3389/fimmu.2025.1512189, 39963134 PMC11830603

[80] Calabresi PA Arnold DL Sangurdekar D Singh CM Altincatal A de Moor C . Temporal profile of serum neurofilament light in multiple sclerosis: implications for patient monitoring. Mult Scler. (2021) 27:1497–505. doi: 10.1177/1352458520972573, 33307998 PMC8414824

[81] Fissolo N Pignolet B Rio J Vermersch P Ruet A de Sèze J . Serum neurofilament levels and PML risk in patients with multiple sclerosis treated with natalizumab. Neurol Neuroimmunol Neuroinflamm. (2021) 8:e1003. doi: 10.1212/NXI.0000000000001003, 33903203 PMC8105883

[82] Quintanilla-Bordás C Gorriz D Cubas-Núñez L Castillo-Villalba J Carreres-Polo J Casanova B . Elevation of serum neurofilament light-chain levels disclose possible occult progressive multifocal leukoencephalopathy and immune reconstitution syndrome in a patient receiving ozanimod: a case report. Front Immunol. (2024) 15:1465678. doi: 10.3389/fimmu.2024.1465678, 39502687 PMC11535851

[83] Bjornevik K Munger KL Cortese M Barro C Healy BC Niebuhr DW . Serum Neurofilament light chain levels in patients with Presymptomatic multiple sclerosis. JAMA Neurol. (2020) 77:58–64. doi: 10.1001/jamaneurol.2019.3238, 31515562 PMC6745051

[84] Jons D Zetterberg H Biström M Alonso-Magdalena L Gunnarsson M Vrethem M . Axonal injury in asymptomatic individuals preceding onset of multiple sclerosis. Ann Clin Transl Neurol. (2022) 9:882–7. doi: 10.1002/acn3.51568, 35502756 PMC9186135

[85] Yusuf FLA Karim ME Sutherland JM Zhu F Zhao Y Marrie RA . Informative patterns of health care utilization preceding the recognition of adult-onset multiple sclerosis: a population-based study. Mult Scler Relat Disord. (2025) 101:106560. doi: 10.1016/j.msard.2025.106560, 40505540

[86] Pawlitzki M Sweeney-Reed CM Bittner D Lux A Vielhaber S Schreiber S . CSF-Progranulin and Neurofilament light chain levels in patients with radiologically isolated syndrome-sign of inflammation. Front Neurol. (2018) 9:1075. doi: 10.3389/fneur.2018.01075, 30619038 PMC6305325

[87] Okuda DT Kantarci O Lebrun-Frénay C Sormani MP Azevedo CJ Bovis F . Dimethyl fumarate delays multiple sclerosis in radiologically isolated syndrome. Ann Neurol. (2023) 93:604–14. doi: 10.1002/ana.26555, 36401339

[88] Norgren N Rosengren L Stigbrand T. Elevated neurofilament levels in neurological diseases. Brain Res. (2003) 987:25–31. doi: 10.1016/S0006-8993(03)03219-0, 14499942

[89] Biernacki T Sandi D Kincses ZT Füvesi J Rózsa C Mátyás K . Emerging biomarkers of multiple sclerosis in the blood and CSF. Int J Mol Sci. (2022) 23:3383. doi: 10.3390/ijms23063383 35328802 PMC8951485

[90] Koerbel K Maiworm M Schaller-Paule M Schäfer JH Jakob J Friedauer L . Evaluating the utility of serum NfL, GFAP, UCHL1 and tTAU as estimates of CSF levels and diagnostic instrument in neuroinflammation and multiple sclerosis. Mult Scler Relat Disord. (2024) 87:105644. doi: 10.1016/j.msard.2024.105644, 38701697

[91] Disanto G Adiutori R Dobson R Martinelli V Dalla Costa G Runia T . Serum neurofilament light chain levels are increased in patients with a clinically isolated syndrome. J Neurol Neurosurg Psychiatry. (2016) 87:126–9. doi: 10.1136/jnnp-2014-309690, 25716934

[92] van der Vuurst de Vries RM Wong YYM Mescheriakova JY van Pelt ED Runia TF Jafari N . High neurofilament levels are associated with clinically definite multiple sclerosis in children and adults with clinically isolated syndrome. Mult Scler. (2019) 25:958–67. doi: 10.1177/1352458518775303 29774770 PMC6545618

[93] Arrambide G Espejo C Eixarch H Villar LM Alvarez-Cermeño JC Picón C . Neurofilament light chain level is a weak risk factor for the development of MS. Neurology. (2016) 87:1076–84. doi: 10.1212/WNL.0000000000003085, 27521440 PMC5027802

[94] Dalla Costa G Martinelli V Sangalli F Moiola L Colombo B Radaelli M . Prognostic value of serum neurofilaments in patients with clinically isolated syndromes. Neurology. (2019) 92:e733–41. doi: 10.1212/WNL.0000000000006902, 30635483 PMC6382362

[95] Saidha S Al-Louzi O Ratchford JN Bhargava P Oh J Newsome SD . Optical coherence tomography reflects brain atrophy in multiple sclerosis: a four-year study. Ann Neurol. (2015) 78:801–13. doi: 10.1002/ana.24487, 26190464 PMC4703093

[96] Solís-Tarazona L Raket LL Cabello-Murgui J Reddam S Navarro-Quevedo S Gil-Perotin S. Predictive value of individual serum neurofilament light chain levels in short-term disease activity in relapsing multiple sclerosis. Front Neurol. (2024) 15:1354431. doi: 10.3389/fneur.2024.1354431, 38426169 PMC10903281

[97] Rolfes L Pawlitzki M Pfeuffer S Nelke C Lux A Pul R . Ocrelizumab extended interval dosing in multiple sclerosis in times of COVID-19. Neurol Neuroimmunol Neuroinflamm. (2021) 8:e1035. doi: 10.1212/NXI.0000000000001035, 34261812 PMC8362352

[98] Piñar-Morales R Calle-Calle R Carrasco-Garcia M Davila-Arias C Villar-Guimerans LM Barrero Hernandez FJ. Case report: Neurofilament light chain in the follow up of progressive multifocal leukoencephalopathy in a patient with multiple sclerosis treated with ocrelizumab. Front Pharmacol. (2025) 16:1571699. doi: 10.3389/fphar.2025.1571699, 40308763 PMC12042224

[99] Cuello JP Martínez Ginés ML Kuhle J García Domínguez JM Lozano Ros A Romero Delgado F . Neurofilament light chain levels in pregnant multiple sclerosis patients: a prospective cohort study. Eur J Neurol. (2019) 26:1200–4. doi: 10.1111/ene.13965, 30977955

[100] Proschmann U Haase R Inojosa H Akgün K Ziemssen T. Drug and Neurofilament levels in serum and breastmilk of women with multiple sclerosis exposed to Natalizumab during pregnancy and lactation. Front Immunol. (2021) 12:715195. doi: 10.3389/fimmu.2021.715195, 34512637 PMC8426350

[101] Proschmann U Inojosa H Akgün K Ziemssen T. Natalizumab pharmacokinetics and -dynamics and serum Neurofilament in patients with multiple sclerosis. Front Neurol. (2021) 12:650530. doi: 10.3389/fneur.2021.650530, 33935948 PMC8079654

[102] Reinert MC Benkert P Wuerfel J Michalak Z Ruberte E Barro C . Serum neurofilament light chain is a useful biomarker in pediatric multiple sclerosis. Neurol Neuroimmunol Neuroinflamm. (2020) 7:e749. doi: 10.1212/NXI.0000000000000749, 32404429 PMC7238898

[103] Huppke B Reinert MC Hummel-Abmeier H Stark W Gärtner J Huppke P. Pretreatment Neurofilament light chain serum levels, early disease severity, and treatment response in pediatric multiple sclerosis. Neurology. (2023) 101:e1873–83. doi: 10.1212/WNL.0000000000207791, 37748882 PMC10663003

[104] Garcia-Dominguez JM Villar LM Arrambide G Blanco Y Calles MC Casanova B . Relevance of serum neurofilament light chain determination as a biomarker in multiple sclerosis. Consensus of the Spanish Society of Neurology's study group on multiple sclerosis and related Neuroimmune diseases. Neurologia. (2026) 41:501928. doi: 10.1016/j.nrl.2025.501928 42044944

[105] Hirt J Janiaud P Düblin P Nicoletti GJ Dembowska K Nguyen TVT . Use of pragmatic randomized trials in multiple sclerosis: a systematic overview. Mult Scler. (2024) 30:463–78. doi: 10.1177/13524585231221938, 38253528 PMC11010556

[106] Janiaud P Zecca C Salmen A Benkert P Schädelin S Orleth A . MultiSCRIPT-cycle 1-a pragmatic trial embedded within the Swiss multiple sclerosis cohort (SMSC) on neurofilament light chain monitoring to inform personalized treatment decisions in multiple sclerosis: a study protocol for a randomized clinical trial. Trials. (2024) 25:607. doi: 10.1186/s13063-024-08454-6, 39261900 PMC11391827

[107] Ontaneda D Tallantyre EC Raza PC Planchon SM Nakamura K Miller D . Determining the effectiveness of early intensive versus escalation approaches for the treatment of relapsing-remitting multiple sclerosis: the DELIVER-MS study protocol. Contemp Clin Trials. (2020) 95:106009. doi: 10.1016/j.cct.2020.106009, 32320842

[108] Mowry EM Qian P Meador W Lynch S Narayan R Borazanci A . The TRaditional versus early aggressive therapy for multiple sclerosis (TREAT-MS) trial: design and baseline characteristics of participants. Contemp Clin Trials. (2025) 159:108117. doi: 10.1016/j.cct.2025.108117, 41135813 PMC12951411

[109] Willard C Puglisi L Ravi D Dmitrieva M Mattiesing RM Barkhof F . Combined magnetic resonance imaging and serum analysis reveals distinct multiple sclerosis types. Brain. (2025) 148:4578–91. doi: 10.1093/brain/awaf331, 41325776 PMC12678051

[110] Martinez-Lapiscina EH Arnow S Wilson JA Saidha S Preiningerova JL Oberwahrenbrock T . Retinal thickness measured with optical coherence tomography and risk of disability worsening in multiple sclerosis: a cohort study. Lancet Neurol. (2016) 15:574–84. doi: 10.1016/S1474-4422(16)00068-5, 27011339

