# Supplementary material for: Serum neurofilament light chain in multiple sclerosis: from biological signal to clinically informed decision-making
Source: Front Neurol. 2026 Jun 24;17:1815022. doi: 10.3389/fneur.2026.1815022 (PMC13341485; doi:10.3389/fneur.2026.1815022)
Supplement: Supplementary file 1 [file Supplementary_file_1.docx]

**SUPPLEMENTARY MATERIAL.**

Supplementary Table S1. High-impact advanced scenarios for sNfL interpretation (decision-support focus)

| **Scenario (decision point)** | **What sNfL adds** | **Typical sNfL pattern** | **Key confounders / pitfalls** | **Practical response (what to do)** | **Key references** |
| --- | --- | --- | --- | --- | --- |
| Clinical/MRI stability (incl. NEDA) but persistently high/rising sNfL | Detects “biology not captured” by routine assessment; prompts structured reappraisal | Sustained elevation or reproducible upward trend | Trauma/stroke; renal dysfunction; metabolic disease; other neurodegeneration; platform inconsistency | Check confounders → repeat sNfL (same platform) → optimize MRI strategy; don’t escalate automatically | 15, 19,62 |
| Anti-CD20 interval extension / de-intensification | Biological surveillance while changing exposure | Low/normal sNfL supports stability; rises raise concern | Over-interpreting single values; comorbidities | Use shared decision; monitor with scheduled MRI+sNfL; treat confirmed rises as “reassess now” | 15,19,99 |
| Early on-treatment monitoring (anti-CD20) to flag inflammatory risk | Identifies early biological non-control despite treatment start | Persistently high z-score early after initiation associated with inflammatory risk | Sampling timing; baseline activity; assay variability | If early elevated: tighten surveillance (MRI timing, clinical review) rather than assume failure on one data point | 7,10,15,84 |
| Natalizumab: differentiate relapse vs PML in a “red-flag” rise | Supports urgency and differential when PML is possible | Markedly higher during PML than relapses; pre-PML signal described | Not specific—can rise in severe relapse/other CNS injury | Urgent MRI + PML work-up when clinical context fits; don’t use as stand-alone diagnosis | 82,83,100 |
| Occult opportunistic disease / PML-IRIS on immunosuppressive DMTs | “Alarm bell” preceding or accompanying imaging change | Unexpected rise months before detection in a case; peaks at presentation | Case-report level evidence; many false positives possible | Treat as trigger for expedited evaluation (neuro exam + MRI; CSF if indicated) | 82,83,100 |
| Pregnancy/postpartum/lactation (MRI constraints; gadolinium avoided) | Adds monitoring channel when imaging is constrained; supports trajectory-based assessment | Pregnancy low; postpartum transient increases may occur even without activity | Parturition effect vs silent activity; single-timepoint misinterpretation | Prefer serial sNfL; confirm rises; integrate with symptoms + non-contrast MRI strategy | 101-103 |
| IRT retreatment decisions (cladribine/alemtuzumab windows) | Flags possible renewed injury during drug-free intervals (supportive, not prescriptive) | High sNfL + MRI activity increases agreement for action in consensus algorithms | Over-reliance without MRI; confounders | Do not retreat on sNfL alone; corroborate with MRI/clinical context; repeat if uncertain | 15,19,80 |
| De-escalation / stopping DMT (older, stable patients) | Supports safer deprescribing framework with tight follow-up | sNfL <80th percentile used as criterion in algorithms | Evidence limited; patient heterogeneity | Only within structured monitoring: 6-monthly MRI+sNfL; re-escalate if activity or high sNfL | 15,19,57,  99 |

Supplementary Table S2. Examples of pragmatic/real-world trials relevant to biomarker implementation in MS

| **Trial** | **Design (pragmatic features)** | **Population** | **Intervention/ strategy** | **Primary outcomes** | **How it informs biomarker implementation** |
| --- | --- | --- | --- | --- | --- |
| MultiSCRIPT-Cycle 1 (106) | Multicenter 1:1 randomized, platform trial embedded in Swiss MS Cohort; routine-data workflows; algorithm-supported decisions | RRMS in cohort ≥1 year | 6-monthly sNfL monitoring + pre-specified action algorithm vs usual care | EDA3 and MSQoL-54 at 24 months | Direct clinical-utility test of sNfL-augmented monitoring and treatment adaptation |
| TREAT-MS (108) | Pragmatic randomized, rater-blinded; clinician–patient chooses DMT within assigned strategy; broad inclusion across US sites | Treatment-naïve RRMS (18–60 years) | Higher-efficacy strategy vs traditional strategy | EDSS-plus sustained worsening | Shows feasibility of strategy-randomization in routine care; infrastructure where biomarkers can be embedded as triggers/stratifiers |
| DELIVER-MS (107) | Pragmatic comparative-effectiveness randomized design with parallel observational cohort; international (US/UK) | Early RRMS | Early highly effective monoclonal antibody strategy vs escalation strategy | MRI brain volume loss to month 36 (primary); disability and other outcomes | Provides a pragmatic framework for testing monitoring/adaptation strategies; biomarkers could complement MRI and support personalization |
